# Integrative analysis of polyamine metabolism-related genes in gliomas: implications for prognosis and therapy

**DOI:** 10.3389/fonc.2025.1517557

**Published:** 2025-07-21

**Authors:** Yujia Zhao, Zhenkai Fu, Sijie Chen, Fei Li, Xiaoyu Zhang, Kaidiriye Setiwalidi, Zhiping Ruan, Yu Yao, Lanxin Luo

**Affiliations:** ^1^ Department of Oncology, Tangdu Hospital, the Fourth Military Medical University, Xi’an, Shaanxi, China; ^2^ Department of Medical Oncology, The First Affiliated Hospital of Xi’an Jiaotong University, Xi’an, China; ^3^ Department of Thoracic Surgery, Peking University People's Hospital, Beijing, China; ^4^ Department of Toxicology, School of Public Health, the Fourth Military Medical University, Xi’an, Shaanxi, China; ^5^ Precision Pharmacy and Drug Development Center, Department of Pharmacy, Tangdu Hospital, the Fourth Military Medical University, Xi’an, Shaanxi, China

**Keywords:** polyamine, glioma, prognosis, immunosuppression, spermine synthase

## Abstract

**Introduction:**

Tumor transformation and progression are accompanied by multiple carcinogenic pathways that dysregulate polyamine demand and metabolism. The importance of polyamines has demonstrated that their metabolism is a potential therapeutic strategy. Yet, few prognostic models based on polyamine metabolism-related gene risk have been developed for gliomas.

**Methods:**

The mRNA expression profiles and variations in 37 polyamine metabolism-related genes (PMRGs) were obtained from the Cancer Genome Atlas (TCGA) and Chinese Glioma Genome Atlas (CGGA) databases. PMRGs-related risk model was constructed by least absolute shrinkage and selection operator (LASSO) Cox regression and tested for predictive ability across two independent datasets from the Gene Expression Omnibus (GEO). The landscape of the tumor immune microenvironment and drug sensitivity were investigated systematically using multiple methods based on PMRG-related risk subtypes. Weighted gene co-expression network analysis (WGCNA) was applied to identify the key prognostic genes of the PMRGs. In addition, key genes were validated with regard to their expression and prognostic significance in human glioma tissues. To verify the cell types, single-cell RNA sequencing was performed on the cohorts available at GEO.

**Results:**

Based on PMRG clusters, patients with glioma showed significant differences in PMRG expression, prognosis, and biological functions. A 11-gene risk model was constructed, and patients were categorized into high- and low-risk subtype according to the risk score. The high-risk subtype exhibited a poorer prognosis due to its immunosuppressive microenvironment. Furthermore, there were striking differences between the distinct subtypes in terms of immune cell infiltration, anticancer immunity cycle, tumor mutation burden, immune checkpoints, and response to targeted inhibitors. Spermine synthase (SMS) was identified as a key PMRG in patients with gliomas. A significant increase in SMS mRNA and protein expression was observed in tumors compared to normal controls. Single-cell sequencing analyses showed that SMS mRNA was highly expressed in all cell types, except oligodendrocytes.

**Conclusion:**

A PMRG-related risk model can be used as a reliable prognostic biomarker in glioma treatment. In addition, polyamine metabolism and function can be successfully targeted therapeutically.

## 1 Introduction

Gliomas are the most malignant tumors of the central nervous system (CNS) (1). The 2021 WHO classification of CNS tumors integrates molecular data for the typing, subtyping, and grading of major tumor groups (2, 3). Histological features have traditionally been used to grade CNS tumors, and certain molecular markers can provide powerful prognostic information (2, 3). Glioblastomas (grade IV, GBM) possess immensely aggressive features, with a median survival time of barely 12–15 months after detection, whereas patients with low-grade gliomas (LGG) have a survival rate of approximately seven years (4). Despite advances in current treatment strategies, gliomas remain highly resistant to interventions. Immunotherapy is highly effective against most malignancies; however, its efficacy remains disappointing for brain tumors owing to their robust immunosuppressive microenvironment (5). Therefore, identification of novel biomarkers for predicting glioma outcomes is critical.

Normal cell functions, including chromatin organization, gene expression regulation, cellular growth and differentiation, and immune processes, require polyamines (6, 7). Dysregulated polyamine metabolism is a hallmark of numerous cancers, with intracellular polyamine homeostasis tightly controlled through synthesis, catabolism, and uptake from the extracellular microenvironment (8, 9). Malignant development of brain tumors, including cell proliferation, growth, migration, and radio-resistance, is inextricably related to polyamine metabolism level (10–13). Notably, Khan et al. demonstrated in preclinical models that dual inhibition of polyamine synthesis and uptake exerts potent cytotoxic effects *in vitro* and prolongs survival in tumor-bearing mice (14). Although experimental evidence is still limited, targeting polyamine metabolism may represent a potential therapeutic strategy for gliomas.

The level of polyamine metabolism can affect immune response in tumor tissues, reduce or inactivate immune cells in the tumor microenvironment (TME), and promote the occurrence of immunologically “cold” tumors (6). Polyamine expression is upregulated in tumor-associated myeloid cells, thus promoting immunosuppression in GBM, whereas polyamine metabolism blockade enhances antitumor immunity and strengthens programmed death 1 (PD-1) immunotherapy efficacy (15). In a study using GBM mouse models, tumor-derived polyamine metabolite spermidine (SPD) was found to accumulate in the TME, promoting protumorigenic characteristics of the immune microenvironment primarily by suppressing the quantity and function of CD8^+^ T cells, thereby enhancing tumor invasiveness. Reducing expression of ornithine decarboxylase (ODC), the rate-limiting enzyme in SPD biosynthesis, did not affect *in vitro* cell growth but significantly prolonged survival in tumor-bearing mice (16). However, the intrinsic relationship between polyamines and glioma immune regulation remains unclear. Herein, we integrated polyamine metabolism-related genes (PMRGs) and identified risk subtypes for the prediction of outcomes and immunotherapy efficacy in patients with glioma using public databases.

## 2 Materials and methods

### 2.1 Data processing

Gene expression and clinical data from the TCGA (https://portal.gdc.cancer.gov/) and CGGA (http://www.cgga.org.cn/) databases were used in the present study and processed further manually using TCGAbiolinks. A total of 1685 patients with glioma and 25 non-tumor cases from four eligible datasets (CGGA-693, CGGA-325, TCGA-LGG, and TCGA-GBM) were included. We first integrated (CGGA-693 and CGGA-325) into a single dataset and (TCGA-LGG and TCGA-GBM) into another, using the R “sva” package. Next, the CGGA and TCGA datasets were integrated into a single dataset (TCGA+CGGA) using the same package and randomly divided equally into a training cohort and an internal validation cohort. We had access to each patient’s RNA-sequencing (RNA-seq) data and follow-up information. The clinicopathological features of the patients are presented in **Supplementary Table S1**. Additionally, two relevant datasets (GSE4412 and GSE43378) were retrieved from the GEO database for additional external validation.

### 2.2 Patient tissue collection

Glioma tissue microarray, including 180 adult glioma specimens (grades I to IV) and four non-tumor brain tissues were collected from Shanghai Zhuoli Biotechnology Co., Ltd. (Shanghai, China) for spermine synthase (SMS) protein expression detection (ZL-XP201402). Six glioma patient tissues and three non-tumor brain tissues were also collected for SMS mRNA expression validation. Sample information is presented in **Supplementary Table S2**.

### 2.3 Somatic mutations and evaluation of copy number variations

Using the “maftools” package in R, differential analysis was performed on the mutations of PMRGs and all genes in different groups. The downloaded data were organized, analyzed and annotated. The “oncoplot” function was used to visualize the calculated gene mutation data, and a waterfall plot was drawn, sorted by gene mutation frequency (17).

### 2.4 Unsupervised clustering of 37 PMRGs

Thirty-seven PMRGs (**Supplementary Table S3**) involved in polyamine biosynthesis, transport, and catabolism were selected (6, 18, 19) for unsupervised clustering using ConsensusClusterPlus on merged TCGA+CGGA RNA-seq data (n = 1,685), normalized to TPM and batch-corrected with ComBat. Clustering used PAM algorithm, 1,000 bootstrap resamples, and Spearman correlation for similarity, determining optimal k = 2 via consensus CDF analysis; cluster stability was validated with consensus matrices (20–22).

### 2.5 Biological functions of 37 PMRGs

Unsupervised non-parametric gene set variation analysis (GSVA) was used to estimate gene set enrichment variation based on 37 PMRG expression profiles. The R “GSVA” package was used to calculate the GSVA score for each sample to quantify the HALLMARK (h.all.v7.5.1.symbols.gmt), Kyoto Encyclopedia of Genes and Genomes (KEGG; c2.cp.kegg.v7.5.1.symbols.gmt), and Gene Ontology (GO; c5.go.v7.5.1.symbols.gmt)-related pathways that were enriched in the samples (23, 24).

### 2.6 PMRG-related risk score construction

Prognostic PMRGs were selected via univariate Cox regression (p < 0.05) and LASSO penalized regression, using 10-fold cross-validation to optimize λ, retaining 11 genes for the risk score formula (Risk score = Σ coefficient × expression) (25, 26). Survival analysis was performed using the Kaplan–Meier method. We compared the ability of the risk score and other clinical indicators to evaluate patient prognosis through receiver operating characteristic (ROC) curve analysis. After combining the risk score with other clinical indicators with good predictive ability, we constructed a nomogram to better reflect the clinical value of the prediction formula.

### 2.7 Tumor immune microenvironment in PMRG-related risk subtypes

Using immune microenvironment-related algorithms, including ESTIMATE (immune/stromal scores) and CIBERSORT(22 immune cell subsets, 1,000 permutations, and a threshold p value of <0.05 was the criterion for the successful computation of a sample), the differences between the groups were compared (27). The ssGSEA algorithm of GSVA package was used to characterize the activity of pathways by calculating the enrichment of collected gene lists. And we used the Tracking Tumor Immunophenotype portal (TIP, http://biocc.hrbmu.edu.cn/TIP/) to analyze the activity of antitumor immune pathway of the Cancer-Immunity Cycle. Additionally, the differences in immune checkpoint gene expression abundance between the groups were calculated. Finally, we evaluated the differences in expression of 27 immune checkpoints (e.g., PD-L1, CTLA4) in different groups.

### 2.8 Therapeutic sensitivity prediction in PMRG-related risk subtypes

Drug sensitivity analysis was performed using data from the Genomics of Drug Sensitivity in Cancer 2 (GDSC2) database (https://www.cancerrxgene.org/). The “oncoPredict” R package was employed to investigate the association between PMRG-related risk scores and drug sensitivity, which was utilized to construct a predictive model linking drug sensitivity to cell line expression profiles. Subsequently, the aforementioned model was applied to estimate the half-maximal inhibitory concentration (IC50) values of chemotherapeutic agents for glioma patients. A Wilcoxon rank-sum test was used to assess the statistical significance of differences between groups.

### 2.9 Identification of key genes

To identify highly correlated gene modules from the polyamine-related genes (HUB genes), we employed Weighted Gene Co-Expression Network Analysis (WGCNA). The R “WGCNA” package was used for sample clustering and removal of outlier data. The appropriate soft threshold weight value was then selected using the “PickSoftThreshold” function, and the hierarchical clustering tree of genes was generated. Branch cutting was performed using the “Dynamic Tree Cut” package. Finally, we estimated the correlation between the module and risk score using two indicators, correlation and p-value, extracted the interested module gene IDs, calculated the intersection with all polyamine-related genes, and obtained the module-polyamine-related gene.

### 2.10 Immunohistochemistry

SMS protein levels were detected using IHC, as previously described (22). Briefly, deparaffinized slides were rehydrated and blocked, and antigen retrieval was performed before incubation with the primary SMS antibody (#824889; ZEN-BIOSCIENCE, Chengdu, China) overnight at 4°C. An HRP-conjugated secondary antibody was then added, and the EnVision Detection Kit (DAKO) was used for visualization. Nuclei were stained with hematoxylin. Following this, the Visiopharm software (Visiopharm, Hørsholm, Denmark) was used to calculate the immunostaining scores.

### 2.11 RNA extraction and RT-PCR

Sangon Biotech (Shanghai, China) provided TRIzol reagent for the extraction of total RNA. Thereafter, the first strand cDNA synthetic kit (Roche, Basel, Switzerland) was used to synthesize the cDNA, as previously described (22).

### 2.12 SMS expression based on single-cell RNA-seq

Data (GSE84465, GSE14882, and GSE141460) from the Gene Expression Omnibus (GEO) repository were used to analyze scRNA-seq. The R “Plot1cell” package was used for cluster analysis on the scRNA-seq data. Analysis was performed using the R “Seurat” package and functions PCElbowPlot, JackStrawPlot, FindAllCluster, DoHeatmap, and RunTSNE. Markers in each cluster were filtered using the FindAllMarkers function in Seurat and used to annotate cell types. The t-SNE map was generated using R packages, including “ggpubr,” “ggthemes,” and “Rtsne.”

### 2.13 Statistical analysis

Statistical analyses were performed in the R statistical environment (version 4.2.3, http://www.r-project.org/). Survival curves were analyzed using Kaplan-Meier survival analysis and a log-rank test. Univariate and multivariate Cox regression analyses were used for prognosis analysis. Correlations were assessed using Spearman’s correlation coefficients. For all comparisons, a two-tailed *p* value < 0.05 was considered statistically significant.

## 3 Results

**Supplementary Figure S1** shows the flowchart for this study.

### 3.1 Genetic landscape of PMRGs in glioma

**Figure 1:**
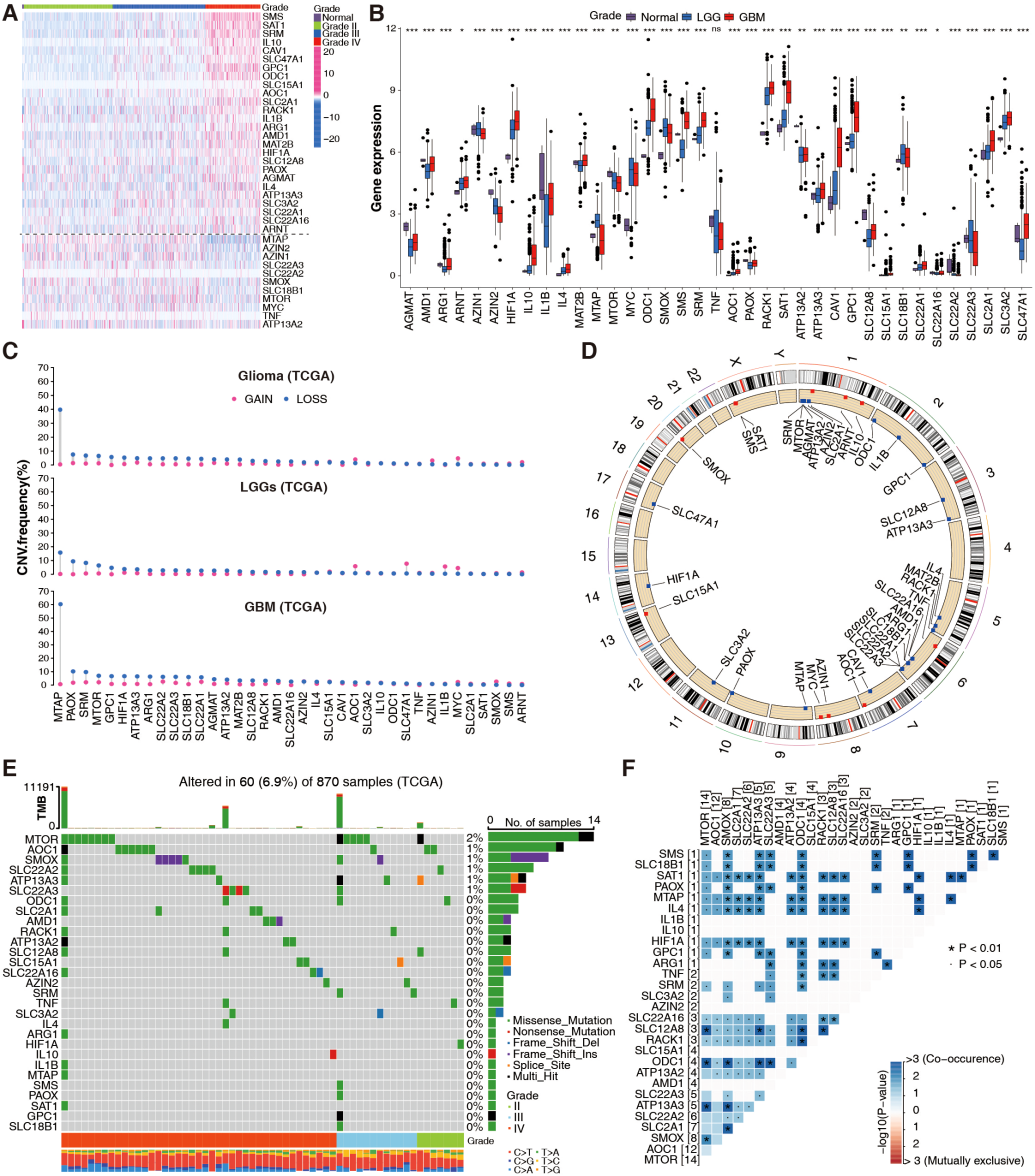
Genetic landscape of PMRGs in the TCGA glioma cohort. **(A)** Differential expression of 37 PMRGs across glioma grades (WHO II–IV) and normal brain tissues (n = 5 normal, n = 667 glioma samples). Heatmaps show log2(TPM + 1) expression; color scale ranges from low (blue) to high (red). **(B)** Differential expression of 37 PMRGs in normal brain tissues, low-grade gliomas (LGG) and GBM; Kruskal-Wallis test, ^*^ *p <*0.05, ^**^ *p <*0.01, ^***^ *p <*0.001. **(C)** Copy number variation (CNV) status of 37 PMRGs, including gain (red) and loss (blue). **(D)** The location of CNVs in chromosomes. **(E)** Somatic mutation profiles of PMRGs in gliomas (n = 870 samples), showing mutation frequency (%) and annotations. **(F)** Co-occurrence analysis of PMRG mutations using Fisher’s exact test (adjusted *p* < 0.05).

Thirty-seven PMRGs related to polyamine metabolism were selected and their expressions were analyzed according to the WHO grade in TCGA cohorts. Most of the selected PMRGs were differentially expressed among LGGs (grades II/III), GBMs, and non-tumor groups (**Figures 1A, B**). Although genes related to polyamine catabolism, such as *SAT1*, *AOC1*, *RACK1*, and *PAOX*, showed the highest expression in GBM tissues and the lowest expression in normal tissues, most of the polyamine biosynthesis-related genes had remarkably increased expression levels in GBM samples, except methylthioadenosine phosphorylase (*MTAP*), *AZIN2*, *AZIN1*, *SMOX*, *MTOR*, and *MYC*. The expression of polyamine transport-related genes increased or decreased with the increasing degree of malignancy (**Figures 1A, B**). There was no significant difference in *TNF* expression (p > 0.05, **Figure 1B**). Thus, patients with glioma can be distinguished based on the expression of PMRGs.

In CNVs, a significant region of tumor DNA is either gained or lost, which may activate oncogenes or inactivate tumor-suppressor genes (28). We calculated frequent alterations in 37 PMRGs, and demonstrated the most significant CNV depletions in the *MTAP* gene for GBM and relatively significant CNV amplification in the *AZIN1*, *MYC*, *AOC1*, and *CAV1* genes for LGG (**Figure 1C**). **Figure 1D** shows the chromosome location and mutation frequency of 37 PMRGs. PMRG mutations were detected in 60 of the 870 samples (mutation rate 6.9%; **Figure 1E**). *MTOR* had the highest mutation frequency, while mutation frequencies for other PMRGs ranged between 0 and 1% (**Figure 1E**). Activating somatic *MTOR* mutations have been identified recently in human cancers (29). The top 20 PMRG mutations more frequently occurred in GBM than in LGG (**Figure 1E**). Additionally, PMRG mutation co-occurrence was more frequently observed in gliomas (**Figure 1F**). Overall, these results suggest that PMRG variation and mRNA expression patterns differ between gliomas of different grades.

### 3.2 Evaluation of prognosis and immune infiltrating patterns with distinct PMRG clusters

**Figure 2:**
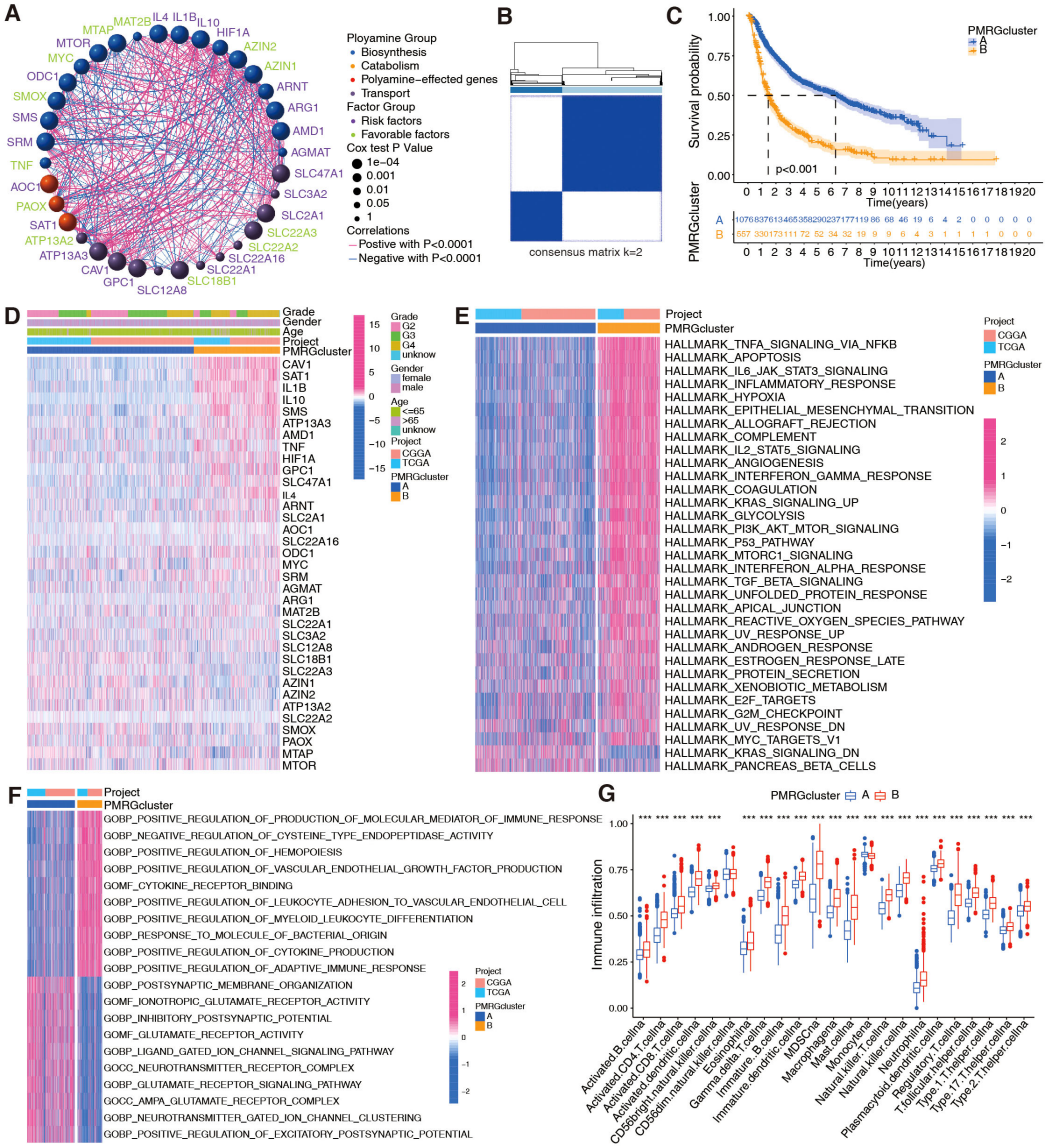
Evaluation of prognosis and biological functions based on PMRG clusters in integrated TCGA+CGGA dataset (n = 1,685 patients). **(A)** Interactions between PMRGs. P-values for prognosis are represented by circle size. Green color represents favorable prognostic factors, and purple color, risk factors. Line thickness indicates correlation strength between PMRGs. Negative and positive correlations are marked in blue and red, respectively. **(B)** Consensus clustering matrix for glioma samples (k = 2) using ConsensusClusterPlus. **(C)** Kaplan-Meier curves for overall survival (OS) between PMRG clusters (Cluster A *vs.* Cluster B); log-rank test, *p* < 0.001. **(D)** Differential PMRG expression between cluster. **(E, F)** HALLMARK and Gene Ontology signature enrichment between PMRG clusters. **(G)** Immune cell infiltration profiles (23 cell types) between clusters, estimated by ssGSEA; Wilcoxon test, ^***^ *p <*0.001.

PMRGs in the TCGA+CGGA cohort were examined for interactions, connections, prognostic impact, and biological functions. **Figure 2A** shows interactions among the three PMRG groups (biosynthesis, metabolism, and polyamine transport). Prognostically favorable PMRGs were considerably negatively correlated with unfavorable PMRGs; for example, the favorable *MTAP* gene had a negative correlation with the unfavorable genes *SLC47A1*, *GPC1*, *SMS*, and *SRM*. The unfavorable prognostic gene *HIF1A* was negatively correlated with the favorable prognostic genes *PAOX* and *ATP13A2* (**Figure 2A**). Cluster analysis based on PMRG expression patterns revealed two phenotypes, polyamine clusters A and B (**Figure 2B**), with cluster A having a better prognosis than cluster B, regardless of cancer grade (**Figure 2C**). The generated heatmap showed that the two clusters exhibited different expression patterns (**Figure 2D**).

Next, the biological functions between the two polyamine clusters were analyzed. HALLMARK and KEGG analyses revealed that patients in cluster B exhibited an enrichment of multiple immune activation pathways, including TNF-alpha signaling via NF-κB, IL-6/JAK/STAT3 signaling, inflammatory response, interferon-gamma response, TGF-beta signaling, and cytokine–cytokine receptor interaction (**Figure 2E**; **Supplementary Figure S2A**). GO analysis revealed positive regulation of the production of molecular mediators of the immune response, myeloid leukocyte differentiation, cytokine production, and adaptive immune response in patients with cluster B gliomas (**Figure 2F**). Since polyamine metabolism is involved in remodeling immune responses in TME (30), we used bioinformatics to explore whether it is correlated with glioma immunity. Analysis of immune cell-infiltration characteristics for clusters A and B (**Figure 2G**) showed significant immune cell infiltration for most immune cells in cluster B, except for CD56dim natural killer cells and monocytes. However, immune capacity was not necessarily enhanced owing to the presence of suppressive factors in the TME (31, 32). Hence, we performed cancer-immunity cycle analysis and observed that various steps in the cycle, including the release of cancer cell antigens (step 1), immune cell recruitment (step 4; except for Th17 and B cell recruitment), and recognition of cancer cells by T cells (step 6), were upregulated in cluster B (**Supplementary Figure S2B**). Cancer antigen presentation (step 2) and priming and activation (step 3) were upregulated in cluster A (**Supplementary Figure S2B**). Importantly, the killing of cancer cells was weaker in cluster B than in cluster A (**Supplementary Figure S2B**).

### 3.3 Construction of the PMRG-related risk score

To accurately group patients with glioma according to prognosis, a risk score was computed using Cox regression and LASSO analyses, and risk score-related genes were identified (**Supplementary Figures S3A, B**). Eleven of the 37 PMRGs were classified as risk score-related (*CAV1*, *SMS*, *SLC47A1*, ornithine decarboxylase 1 [*ODC1*], *MTOR*, *GPC1*, *IL10*, *AMD1*, *AOC1*, *MTAP*, and *SLC18B*). Four genes (*CAV1*, *GPC1*, *SLC47A1*, and *SLC18B1*) were related to polyamine transport, one (*AOC1*) to catabolism, and the other six genes were related to polyamine biosynthesis. Prognostic performance was assessed using a polyamine-related prognostic signature. A prediction model was built by combining the expression levels of these 11 prognostic genes, and the patients were divided into high- and low-risk groups according to the following formula:

Risk score = (0.0894 * *CAV1*) + (0.2297 * *IL10*) + (0.2444 * *SMS*) + (0.1675 * *AMD1*) + (0.1216* *GPC1*) + (0.4173 * *SLC47A1*) + (0.5484 * *AOC1*) + (0.2502 * *ODC1*) + (-0.1095 * *SLC18B1*) + (-0.1740 * *MTAP*) + (0.3995 * *MTOR*).

**Figure 3:**
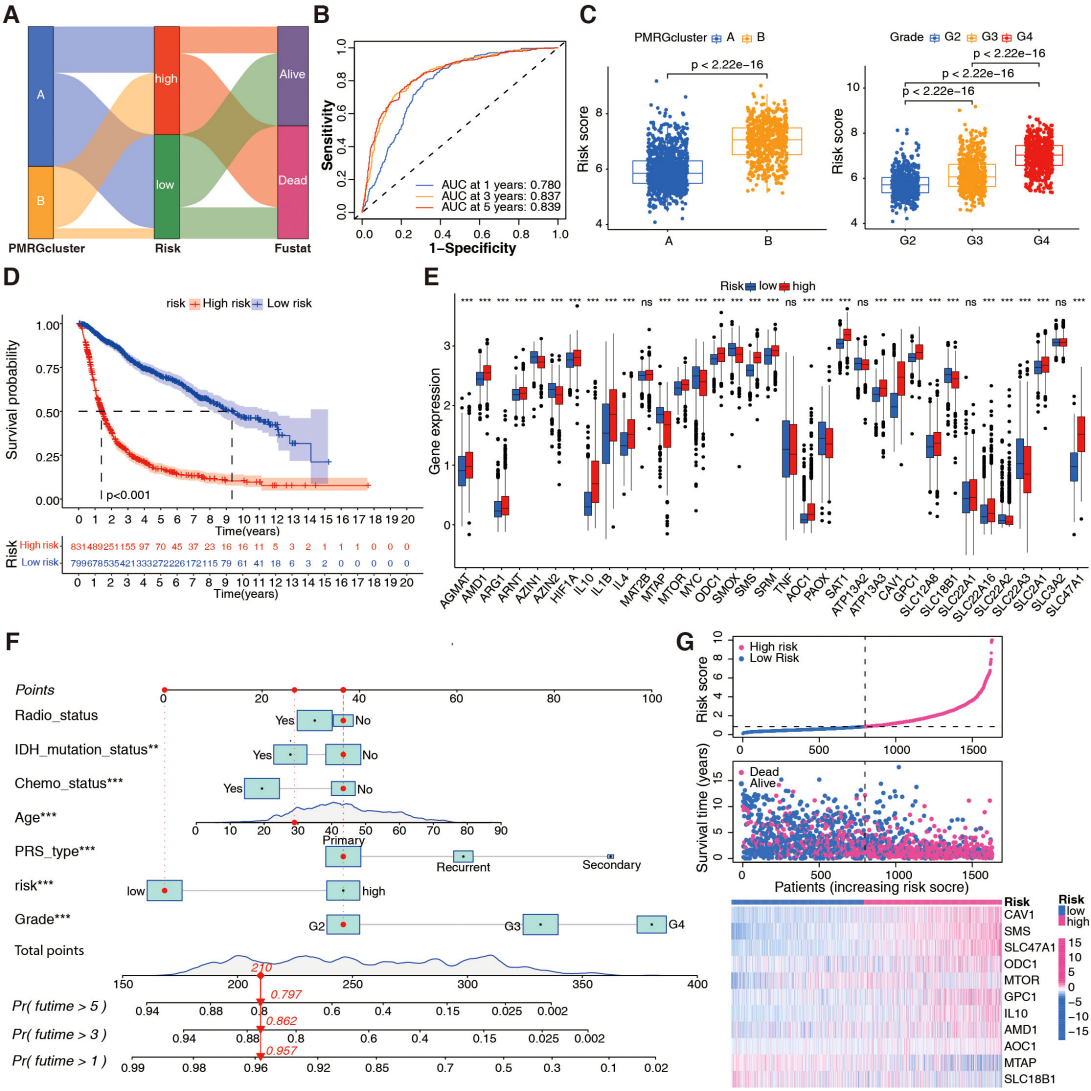
Development of the PMRG-related risk score in the integrated TCGA+CGGA dataset. **(A)** Sankey diagram illustrating distributions of PMRG clusters **(A, B)**, risk score tertiles (low/high), and patient statuses (alive/dead). **(B)** Time-dependent receiver operating characteristic (ROC) curve for OS prediction at 1/3/5 years; area under curve (AUC) = 0.780/0.837/0.839. **(C)** Risk score distribution across PMRG clusters (Wilcoxon test) and glioma stages (WHO II–IV; Kruskal-Wallis text). **(D)** Kaplan-Meier curves for risk subtypes (low vs. high); log-rank test, *p* < 0.001. **(E)** Differential expression of PMRGs between risk subtypes; Wilcoxon test, ^***^ *p <*0.001. **(F)** Nomogram integrating age, WHO grade, risk score, etc. to predict 1, 3, 5-year OS probability; calibration curve (inset) shows agreement between predicted and observed survival. **(G)** Expression heatmap of 11 prognostic genes ordered by increasing risk score.

To further identify the effects of PMRGs on patients with glioma based on risk scores, Sankey plots were used to visualize polyamine cluster distributions, PMRG-related risk scores, and patient status. Most patients in polyamine cluster A had low risk scores and good prognoses (**Figure 3A**). PMRG-related risk scores showed good predictive power (**Figure 3B**). The PMRG-related risk score was also analyzed in the TCGA+CGGA cohort in relation to polyamine clusters, pathological grade, isocitrate dehydrogenase (IDH) status, 1p/19q codeletion, MGMT methylation, age, gender, and polygenic risk score type. Interestingly, patients with grade IV glioma, wild-type IDH, no co-deletion of 1p/19q, and older (>41 years) had a high-risk score in all three datasets, indicating that a high-risk score was likely to predict malignancy (**Figure 3C**; **Supplementary Figures S3C–H**). Regarding the prognostic value of distinct risk-score groups, patients in the TCGA+CGGA cohort with high-risk gliomas, regardless of grade, had poor prognoses (**Figure 3D**). A plot of the 37 PMRG expression levels in the distinct risk-score groups is shown in **Figure 3E**. Based on variable (gender, age, risk score, and disease stage) values, a nomogram was designed to predict overall survival (**Figure 3F**). A risk score-based expression analysis of these 11 genes is illustrated in **Figure 3G**. Moreover, we evaluated the prognostic classification performance of the risk score in the TCGA internal validation cohort and two independent datasets, GSE4412 and GSE43378 (**Supplementary Figure S4**). It is evident that the model shows high robustness and accuracy. Overall, patients with glioma can be classified into distinct risk subtypes based on PMRG expression.

### 3.4 Assessment of immune characterization with PMRG-related risk score

**Figure 4:**
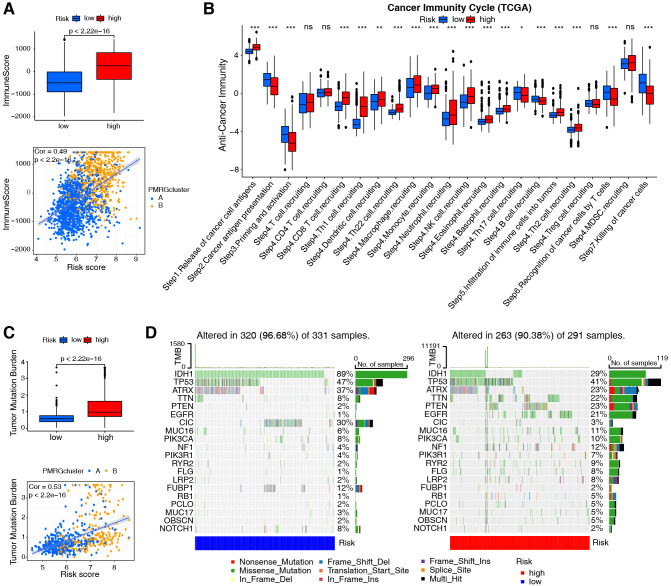
Evaluation of immune cell infiltration and tumor mutation burden based on PMRG-related risk subtypes. **(A)** Immune scores (ESTIMATE algorithm) between low- and high-risk subtypes; Wilcoxon test (*p*-values shown). Correlation between risk score and immune scores; Pearson test (Cor and *p*-values shown). **(B)** Difference between low- and high-risk groups at distinct stages of the cancer-immune cycle; Wilcoxon test, ^*^ *p <*0.05, ^**^ *p <*0.01, ^***^ *p <*0.001. **(C)** TMB comparison between risk subtypes; Wilcoxon test (*p*-values shown). Correlation between risk score and TMB; Pearson test (Cor and *p*-values shown). **(D)** Top 20 most frequently mutated genes in low- and high-risk subtypes (n = 622 TCGA samples), with mutation frequencies (%) indicated.

Immune characterization was performed using the integrated TCGA+CGGA dataset according to different PMRG-related risk groups. An association was identified between PMRG-related risk and immune score (R = 0.49; **Figure 4A**). By combining the two datasets, a strong correlation was found between the PMRG-related risk score and M0 macrophages (R = 0.33), M2 macrophages (R = 0.29), monocytes (R = -0.29), and memory B cells (R = -0.26; **Supplementary Figure S5A**). **Supplementary Figure S5B** shows that immune cell infiltration is associated with each risk gene. The high-risk groups exhibited significantly high expression of *CAV1*, *SMS*, and *SLC47A1*, which were positively correlated with M2 and M0 macrophages, neutrophils, CD8 T cells, and gamma delta T cells, and negatively correlated with activated mast cells, monocytes, CD4 memory resting T cells, and CD4 naïve T cells. Conversely, the low-risk groups had high levels of *MTAP* and *SLC18B1*, which were positively associated with monocytes and memory resting T cells and negatively associated with M0 macrophages. Additionally, analysis of cancer-immunity cycles showed increased release of cancer cell antigens (step 1) and immune cell recruitment (step 4), and decreased release of cancer antigen presentation (step 2), priming and activation (step 3), and killing of cancer cells (step 7; **Figure 4B**) in the high-risk groups.

Subsequently, assessment of the tumor mutation burden (TMB) in TCGA cohorts revealed that patients with high-risk scores had higher TMB than those with low-risk scores (R = 0.53; **Figure 4C**). Cancers with high TMB are more likely to activate the immune system than those with low TMB (33). However, a high TMB is not a reliable predictor of immune checkpoint blockade (ICB) responses across all cancer types (34). We also evaluated somatic mutations associated with the top 20 most frequently mutated driver genes and found that low-risk individuals were more likely to carry *IDH1*, *CIC*, and *FUBP1* mutations, while high-risk individuals were more likely to carry *TTN*, *PTEN*, and *EGFR* mutations (**Figure 4D**).

**Figure 5:**
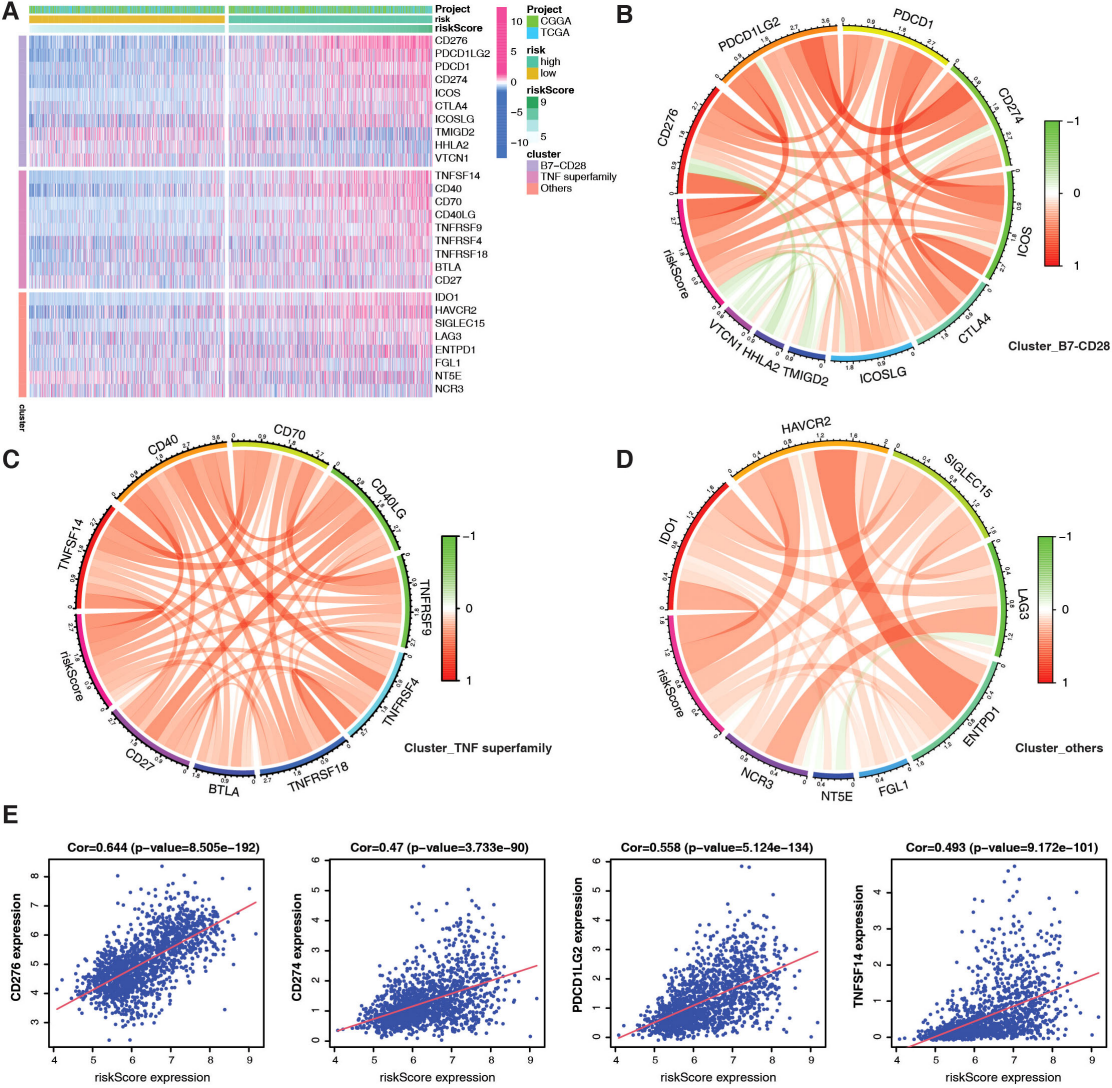
Evaluation of immune checkpoints based on PMRG-related risk subtypes. **(A)** Different expression of immune checkpoints between PMRG-associated risk subtypes. **(B–D)** Correlation between immune checkpoint members of the TNF superfamily **(B)**, the B7-CD28 superfamily **(C)**, and others **(D)**. **(E)** Correlation between specific immune checkpoint members and risk scores; Pearson test (Cor and *p*-values shown).

TMB is a potential biomarker for identifying patients with cancer who may benefit from ICB. Thus, we examined 27 immune checkpoints, including the B7-CD28 and TNF superfamilies, in the TCGA+CGGA dataset. Most immune checkpoint molecules, such as *CD274* (PD-L1), *PDCD1* (PD-1), and *TNFSF14*, were highly expressed in the high-risk group (**Figure 5A**). **Figures 5B–D** depicts the correlation between each family, with PMRG-related risk scores positively correlating with *CD276* (R = 0.644), *CD274* (R = 0.47), *PDCD1LG2* (R = 0.558), and *TNFSF14* (R = 0.493) expression (**Figure 5E**). Overall, the high-risk PMRG subgroup is associated with greater immunosuppression in patients with glioma due to high M2 macrophage infiltration of and high expression of immune checkpoint molecules.

### 3.5 Assessment of chemotherapy efficacy based on PMRG-related risk score

**Figure 6:**
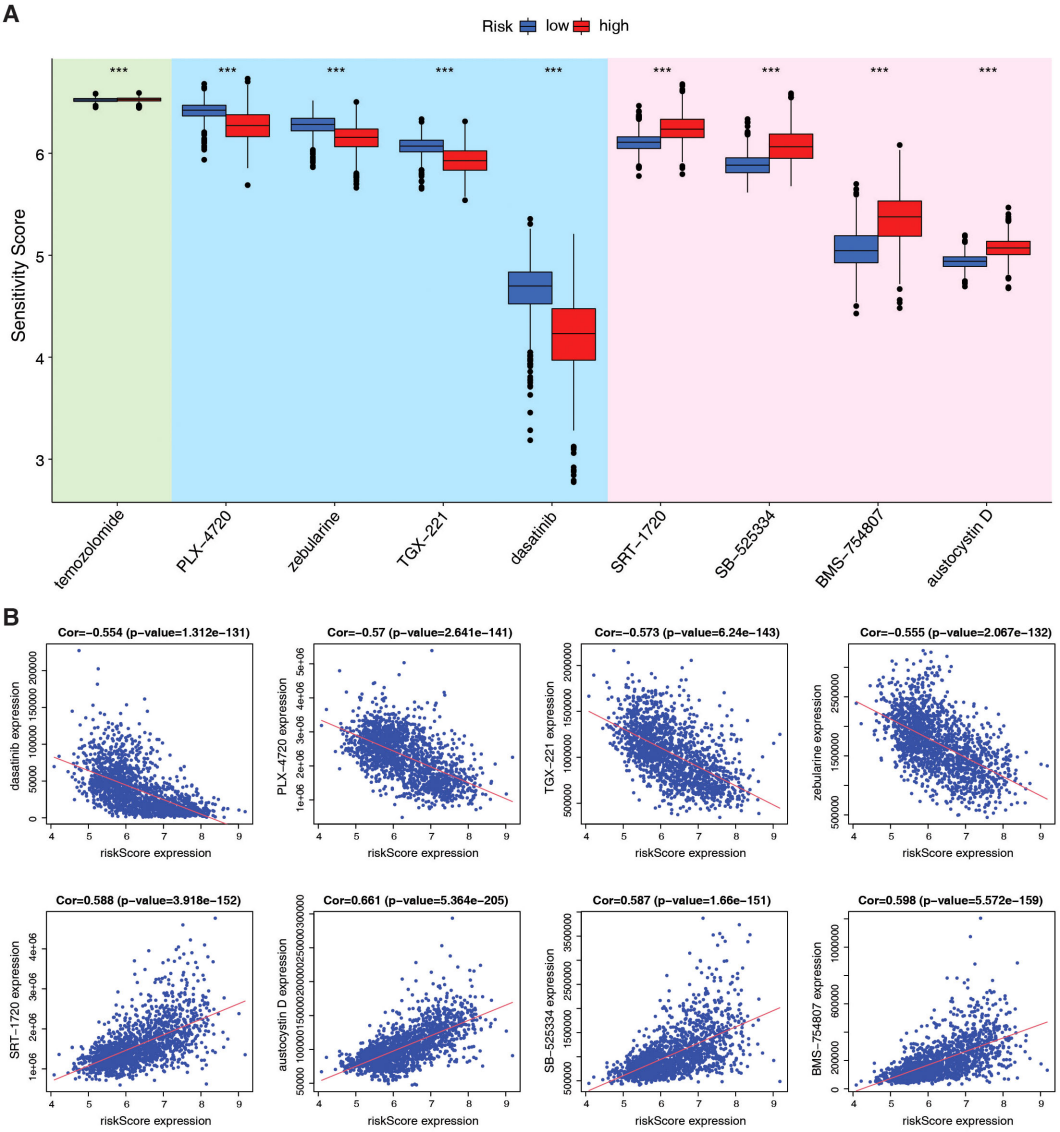
Evaluation of therapeutic efficacy based on PMRG-related risk subtypes. **(A)** Drug sensitivity of 9 chemotherapy agents between risk subtypes, estimated by pRRophetic; Wilcoxon test, ^***^ *p <*0.001. **(B)** Correlation between agents and risk scores; Pearson test, (Cor and *p*-values shown).

Due to the unsatisfactory efficacy of immunotherapy in patients with brain tumors, we analyzed chemotherapy efficacy based on the PMRG-related risk score to explore the potential of drug combinations. To determine whether the risk score could predict clinical therapeutic efficacy in gliomas, we assessed the sensitivity of several agents from the Cancer Therapeutics Response Portal (CTRP) in the TCGA+CGGA dataset using the alkylating agent, temozolomide (TMZ) (35), as a control standard. Chemotherapies were screened based on criteria, including drug sensitivity correlation with risk score, differential drug sensitivity between high- and low-risk score groups, and log2FC value. The top eight therapeutic drugs were more effective than TMZ, with four drugs (dasatinib, PLX-4720, zebularine, and TGX-221) more sensitive in high-risk subtypes and four (SRT-1720, SB-525334, BMS-754807, and austocysin D) in low-risk subtypes (**Figures 6A, B**). These findings suggest that ICB in combination with these chemotherapeutic drugs may be a promising treatment for patients with glioma. While our computational analysis establishes a robust association between PMRG risk scores and chemotherapeutic sensitivity, experimental validation of these predictions, such as drug response assays in glioma cell lines or xenograft models, represents an important avenue for future research.

### 3.6 The *SMS* gene was identified as a prognostic key factor

**Figure 7:**
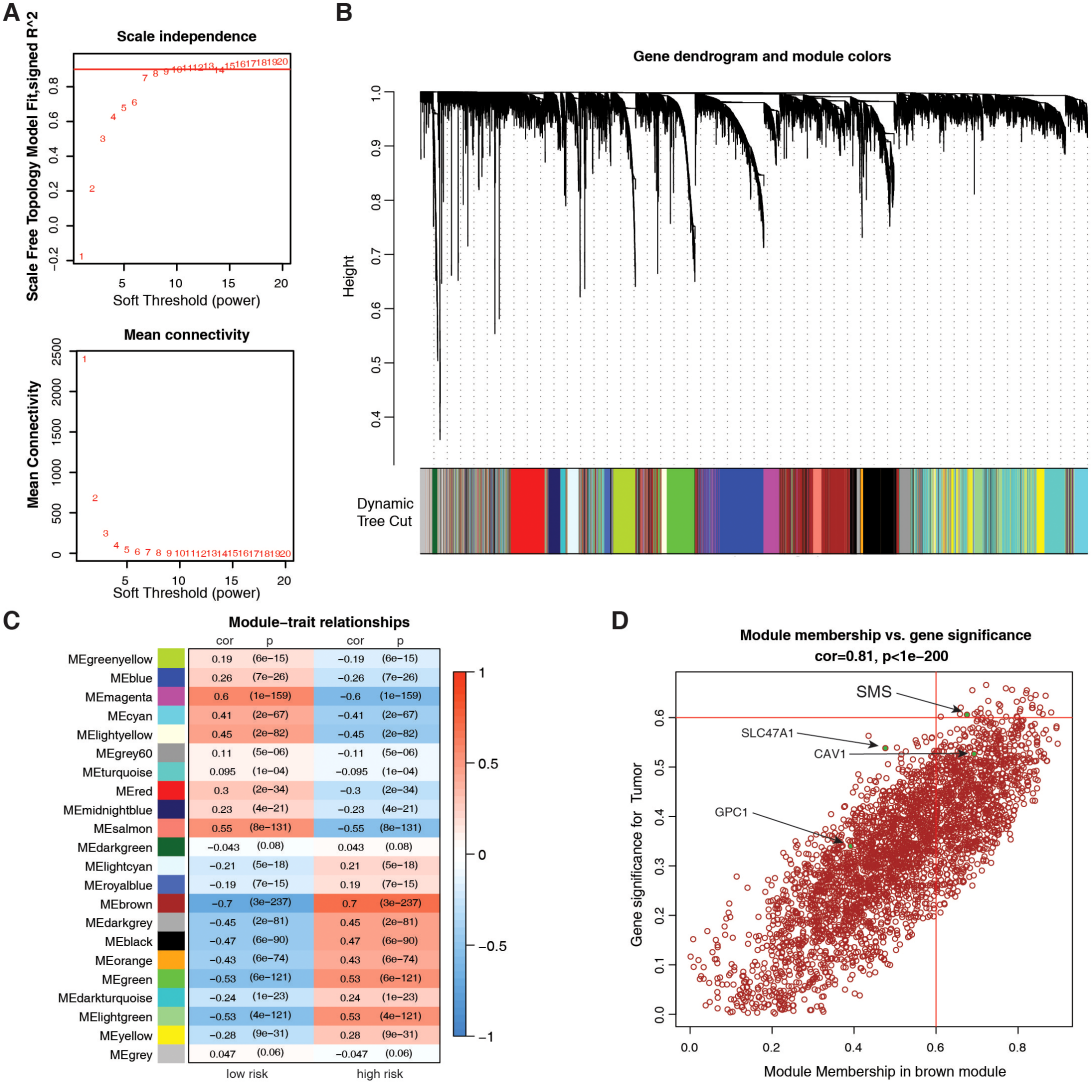
Identification of key PMRG via weighted gene co-expression network analysis (WGCNA). **(A)** Scale-free topology validation for co-expression network construction (soft-thresholding power β = 8). **(B)** Dendrogram of 22 gene modules (average linkage hierarchical clustering) in the glioma transcriptome. **(C)** Module-trait correlation for risk score; color = Pearson correlation coefficient (cor), text = *p*-value. **(D)** Scatter plot of the brown module membership vs. gene significance; Pearson test, (Cor and *p*-values shown).

To identify genes that correlate most strongly with PMRG-related risk scores, we built a gene co-expression network using WGCNA. We constructed a scale-free co-expression network with eight soft thresholds (**Figure 7A**), and 22 modules were established (**Figure 7B**). **Figure 7C** shows that the risk score was most positively correlated with the brown module. By crossing the 11 prognostic genes with the brown module genes, we identified *SMS* as the most significant prognostic gene (**Figure 7D**).

**Figure 8:**
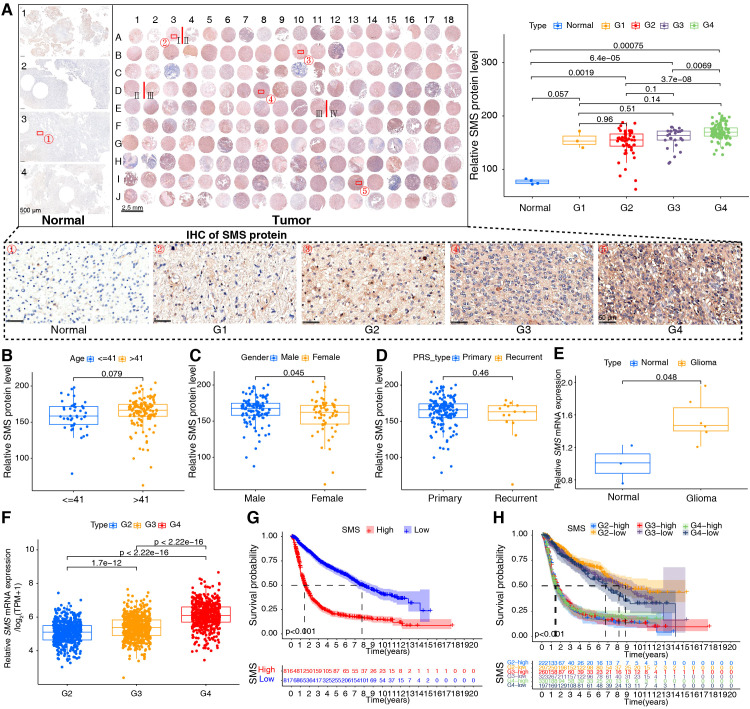
Validation of spermine synthase (SMS) in gliomas. **(A)** SMS protein expression in human tissues; Kruskal-Wallis test (*p*-values shown). **(B–D)** SMS protein levels stratified by age (≤41 vs. >41 years), gender (male vs. female), and tumor status (primary vs. recurrent); Wilcoxon test (*p*-values shown). **(E)** SMS mRNA expression in normal (n = 3) and glioma (n = 6) tissues; Wilcoxon test (*p*-values shown). **(F)** SMS mRNA across glioma grades (WHO I–IV) in the integrated dataset; Kruskal-Wallis test (*p*-values shown). **(G, H)** Kaplan–Meier curves showing overall survival differences between high- and low-SMS expression groups in the integrated dataset; log-rank test, *p* < 0.001 for all.

**Figure 9:**
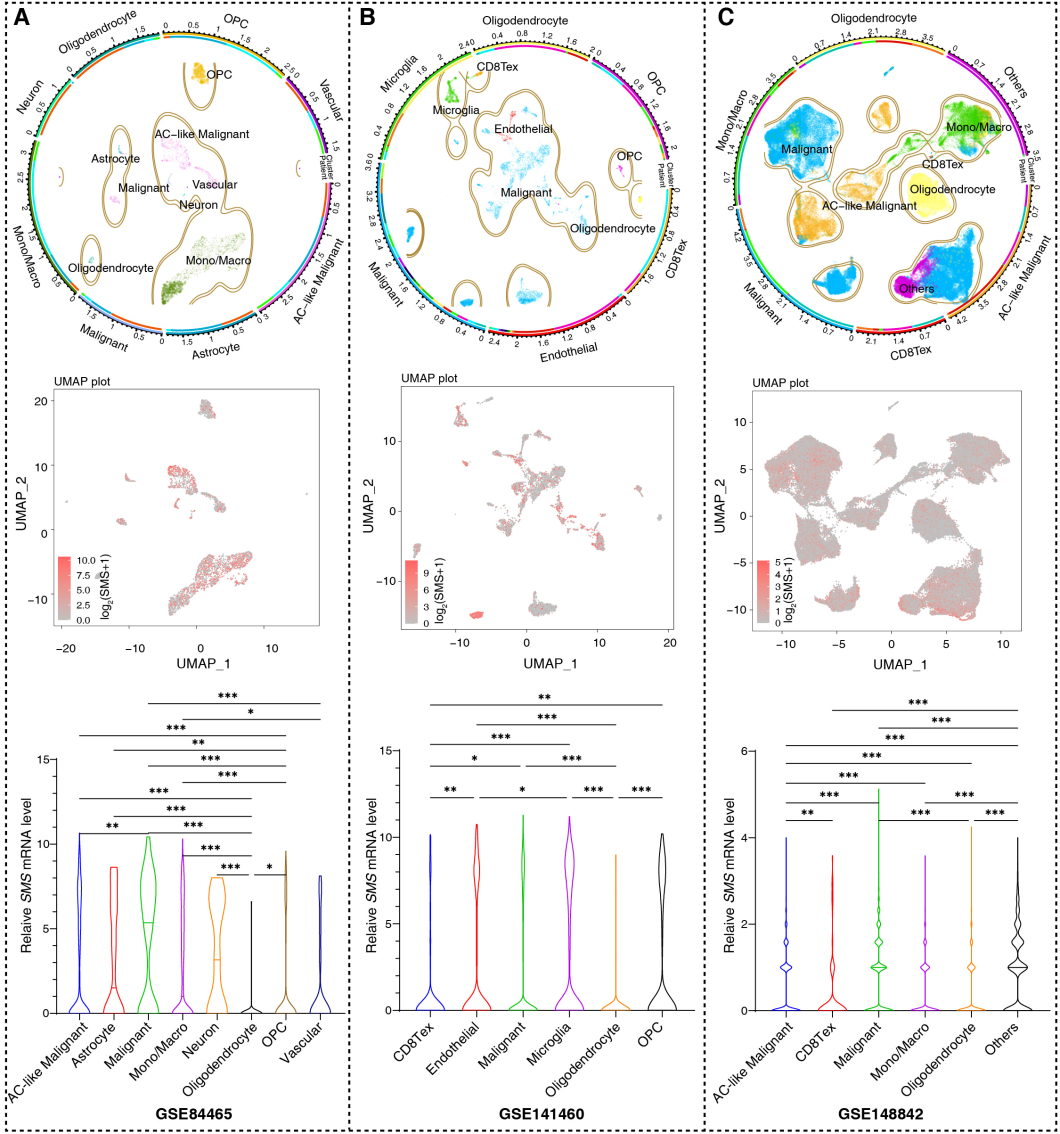
SMS expression based on single cell RNA sequencing. **(A–C)**. Uniform Manifold Approximation and Projection (UMAP) plots from three datasets (GSE84465, GSE14882, GSE141460), annotated by cell types (top panels) and SMS expression (red = positive, gray = negative; intermediate panels). Bottom panels: statistical summaries of SMS positive cell proportions per cell type; Kruskal-Wallis test, ^*^ *p <*0.05, ^**^ *p <*0.01, ^***^ *p <*0.001.

Based on our analysis in human glioma tissues and the TCGA+CGGA dataset, SMS protein and mRNA expression were higher in tumor than in non-tumor tissues and positively correlated with tumor grades (**Figure 8A**). Moreover, SMS protein expression was slightly higher in older groups (>41 years), but the difference was not significant (**Figure 8B**). SMS protein expression showed no significant changes according to sex (**Figure 8C**) and in primary recurrence samples (**Figure 8D**). *SMS* mRNA expression, verified in glioma tissues (**Figure 8E**) and datasets (**Figure 8F**), was higher in tumor than in non-tumor tissues and positively correlated with tumor grades. The prognostic analysis to evaluate the significance of *SMS* in glioma tumors (**Figures 8G, H**) showed that patients with higher SMS expression had a poorer prognosis, regardless of tumor grade. In addition, three single-cell transcriptome datasets (GSE84465, GSE141460, GSE148842) were selected to better study the impact of *SMS* gene expression on the TME. As shown in **Figures 9A–C**, *SMS* was expressed in almost all cell types, including tumor cells, neurons, monocytes/macrophages, astrocytes, oligodendrocytes, and CD8 T cells, suggesting that SMS may affect TME. These findings indicate that SMS overexpression may play an important role in glioma progression, particularly in the formation of an immunosuppressive microenvironment, providing insight into immunotherapeutic strategies for glioma.

## 4 Discussion

Gliomas comprise >70% of malignant primary brain tumors in adults, with GBM being the most common and aggressive, having a five-year survival rate of 4.7% (36, 37). Although tremendous effort has been made to improve therapeutic approaches for gliomas, resection and radio/chemotherapy remain the standard of care (38). The TME, which contains various non-cancer cell types, limits glioma therapy (39). Immunotherapy has shown positive responses in various solid tumors, but patients with glioma are highly resistant to ICB therapies (40, 41). Therefore, improving the efficacy of immunotherapy for gliomas with highly immunosuppressive TMEs is urgent.

Polyamines, which are widely present in mammalian cells, create a tumor-permissive microenvironment (6, 14, 42, 43). Polyamines regulate the anti-tumor immune response, leading to the development of immunologically ‘‘cold’’ tumors that do not respond to ICB (30). However, excessive polyamine metabolism and elevated spermine and spermidine levels in malignant tumors have been associated with immunosuppressive phenotypes. Polyamines are essential for supporting the growth and metabolism of cells that display immunosuppressive phenotypes (44–46). For instance, polyamines suppress lymphocyte proliferation, reduce neutrophil locomotion and NK cell activity, and reprogram macrophages from pro-inflammatory M1 to anti-inflammatory M2 phenotypes (30). Myeloid-derived suppressor cells and dendritic cells also rely on polyamine metabolism to activate their suppressive activities (47). Thus, polyamine-targeting may enhance ICB efficacy by alleviating the effects of immunosuppressive TMEs. We conducted a comprehensive analysis of PMRGs within the glioma microenvironment to provide promising therapeutic intervention strategies.

Analysis of the expression patterns of 37 PMRGs in glioma tumors, revealed that different grades of gliomas could be distinguished from normal cases based on PMRG expression. Evaluation of CNV of PMRGs in glioma tumors identified significant depletion in *MTAP*. Protein arginine methyltransferase 5 (PRMT5) is inhibited by high levels of MTA, rendering PRMT5-deficient cells more susceptible to PRMT5 inhibitors (48). MTAP is essential in polyamine biosynthesis and catalyzes the conversion of MTA to methionine and adenine. Homozygous deletion of MTAP is one of the most frequent genetic alterations in GBM. MTAP deficiency leads to epigenetic reprogramming, promotes the formation of glioma stem-like cells, increases PROM1/CD133 expression and tumor incidence, and is associated with poor patient prognosis (49).

Predictions for patients with gliomas could be obtained using the 37 PMRG signatures. Patients in polyamine metabolism cluster A had a better prognosis than those in cluster B, suggesting that PMRG signatures are good predictors of gliomas. Analysis of biological functions based on the polyamine clusters showed that most processes were related to the immune response. Patients in cluster B showed a high infiltration of most immune cells; however, the killing of cancer cells was weaker in cluster B than in cluster A. This may be due to the production of immunosuppressive factors and/or high levels of immune checkpoints in the TME (20).

We constructed a prognostic signature based on 11 polyamine-related genes and divided patients into PMRG-related high- and low-risk groups. Among the identified genes, *AOC1* encodes a copper-containing amine oxidase that catalyzes the deamination of polyamines to produce reactive oxygen species (50), AOC1 is highly expressed and contributes to tumor progression in various tumors, including gastric, prostate, and colorectal cancers, by promoting the AKT, EMT, and JAK/STAT3 signaling pathways (50–53). However, the effect of AOC1 on glioma progression remains unclear. *SLC47A1*, *GPC1*, *CAV1*, and *SLC18B1* are involved in polyamine transport; however, only *CAV1* has been extensively studied in gliomas and is involved in stemness and temozolomide resistance (54). Among other polyamine biosynthesis-related genes, *ODC1*, the rate-limiting enzyme in polyamine synthesis, is critically associated with the oncogenesis of diffuse intrinsic pontine glioma and neuroblastoma and is therefore a potential therapeutic target (14, 55).

Immune cell infiltration analysis showed that these 11 genes were negatively related to memory B and activated NK cells and positively associated with M2 macrophages and regulatory T cells, which explains why PMRGs promote an immunosuppressive microenvironment. According to the cancer-immunity cycle analysis, high-risk individuals had a significant reduction in their ability to kill tumor cells. These results are consistent with those in published literature (56). Analysis of immune checkpoint molecules showed that the expression levels of *CD276*, *PD-L1*, *PD-1*, *TNFSF14*, and *IDO1* were upregulated as the PMRG-related risk score increased. However, the relationship between polyamine metabolism and immune checkpoints has not yet been elucidated.

Among adverse prognostic regulators, *SMS* overexpression in gliomas was related to poor prognosis. Overexpression of SMS in liver, head and neck, and colon cancers has been associated with a poor prognosis; however, the underlying mechanism remains to be elucidated (57–59). scRNA-seq analysis showed that *SMS* was expressed in all cell types in TME, indicating the importance of SMS in polyamine metabolism. Nevertheless, the potential role of SMS in glioma development and progression requires further investigation.

## Data Availability

The data presented in the study are deposited in the TCGA, CGGA and GEO repositories, with GEO accession numbers GSE4112, GSE43378, GSE84465, GSE14882, and GSE141460.

## Supplementary material

Supplementary Figure 1 Flowchart of the study. Data of 1710 samples from TCGA and CGGA cohorts were integrated, and variations in PMRGs were explored. Patients with glioma were divided into two clusters according to their PMRG-expression profile. Differences in prognosis and biological functions were investigated. To screen the most prognostic-related genes, univariate Cox regression and LASSO analysis were performed, and the patients were divided into PMRG-related risk subtypes. Immune landscapes, including tumor mutation burden, immune cell infiltration, cancer-immunity cycle, immune checkpoint expression, and sensitivity to therapies, were tested in distinct risk subtypes. Key PMRGs that associated with prognosis in glioma tumors were identified and validated in published datasets and human tissues.

Supplementary Figure 2 Evaluation of biological functions based on PMRG clusters in integrated TCGA+CGGA dataset. **(A)** KEGG signature enrichment between PMRG clusters. **(B)** Correlation between the PMRG clusters and cancer immunity cycles.

Supplementary Figure 3
**(A, B)** LASSO coefficient profiles of the 37 PMRGs. **(C–H)** The risk score for glioma patients based on their ages **(C)**, gender **(D)**, PRS types **(E)**, MGMT methylation status **(F)**, 1p19q codeletion status **(G)** and IDH mutation status **(H)**.

Supplementary Figure 4 Validation of the PMRG-related risk score in different cohorts. **(A–C)** ROC curves of the risk scores in the TCGA validation set, GSE4412, and GSE43378; **(D–F)** Kaplan–Meier curves of PMRG-related risk groups in the TCGA validation set, GSE4412, and GSE43378; **(G–I)** Risk score scatter plots of the TCGA validation set, GSE4412, and GSE43378.

Supplementary Figure 5 Evaluation of immune cell infiltration based on PMRG-related risk subtypes. **(A)** Correlation matrix of 22 immune cell infiltrates with risk scores. **(B)** Association of five key PMRGs with immune cell infiltration.
