# Supplementary figures and images for: Integrative analysis of polyamine metabolism-related genes in gliomas: implications for prognosis and therapy
Source: Front Oncol. 2025 Jul 21;15:1517557. doi: 10.3389/fonc.2025.1517557 (PMC12319057; doi:10.3389/fonc.2025.1517557)

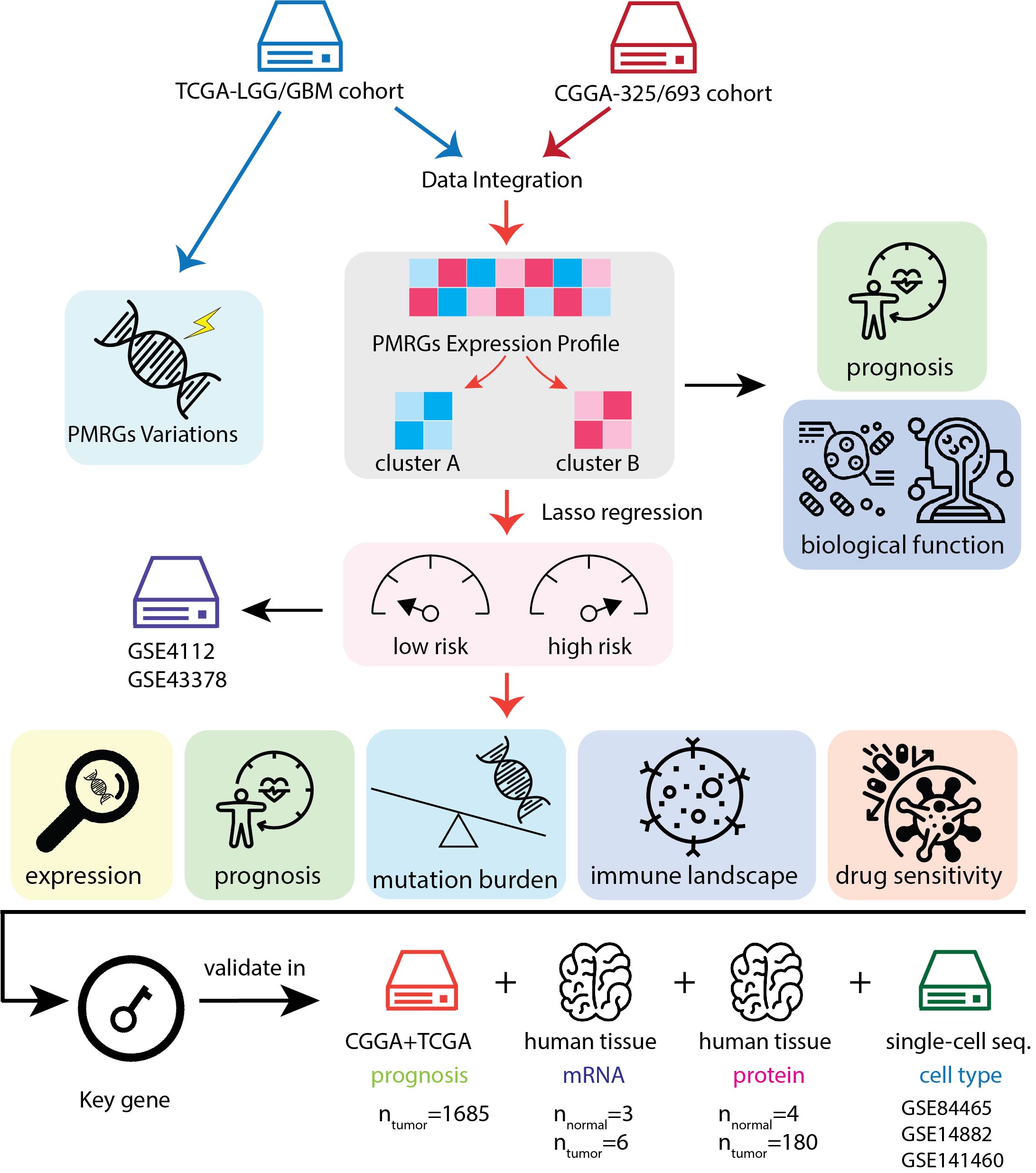

Supplement: Supplementary Figure 1 — Flowchart of the study. Data of 1710 samples from TCGA and CGGA cohorts were integrated, and variations in PMRGs were explored. Patients with glioma were divided into two clusters according to their PMRG-expression profile. Differences in prognosis and biological functions were investigated. To screen the most prognostic-related genes, univariate Cox regression and LASSO analysis were performed, and the patients were divided into PMRG-related risk subtypes. Immune landscapes, including tumor mutation burden, immune cell infiltration, cancer-immunity cycle, immune checkpoint expression, and sensitivity to therapies, were tested in distinct risk subtypes. Key PMRGs that associated with prognosis in glioma tumors were identified and validated in published datasets and human tissues. [file Image1.jpeg]

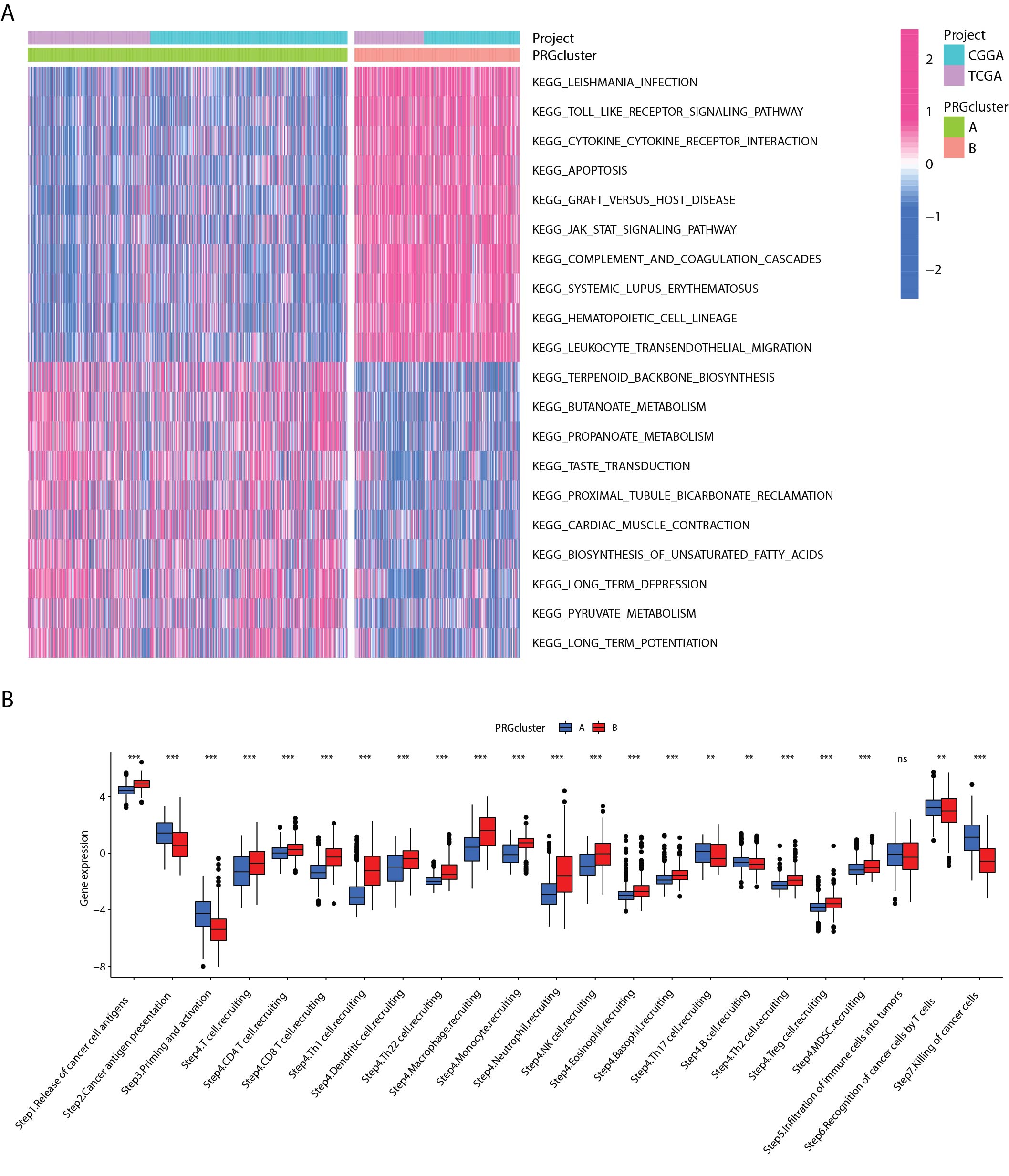

Supplement: Supplementary Figure 2 — Evaluation of biological functions based on PMRG clusters in integrated TCGA+CGGA dataset. (A) KEGG signature enrichment between PMRG clusters. (B) Correlation between the PMRG clusters and cancer immunity cycles. [file Image2.jpeg]

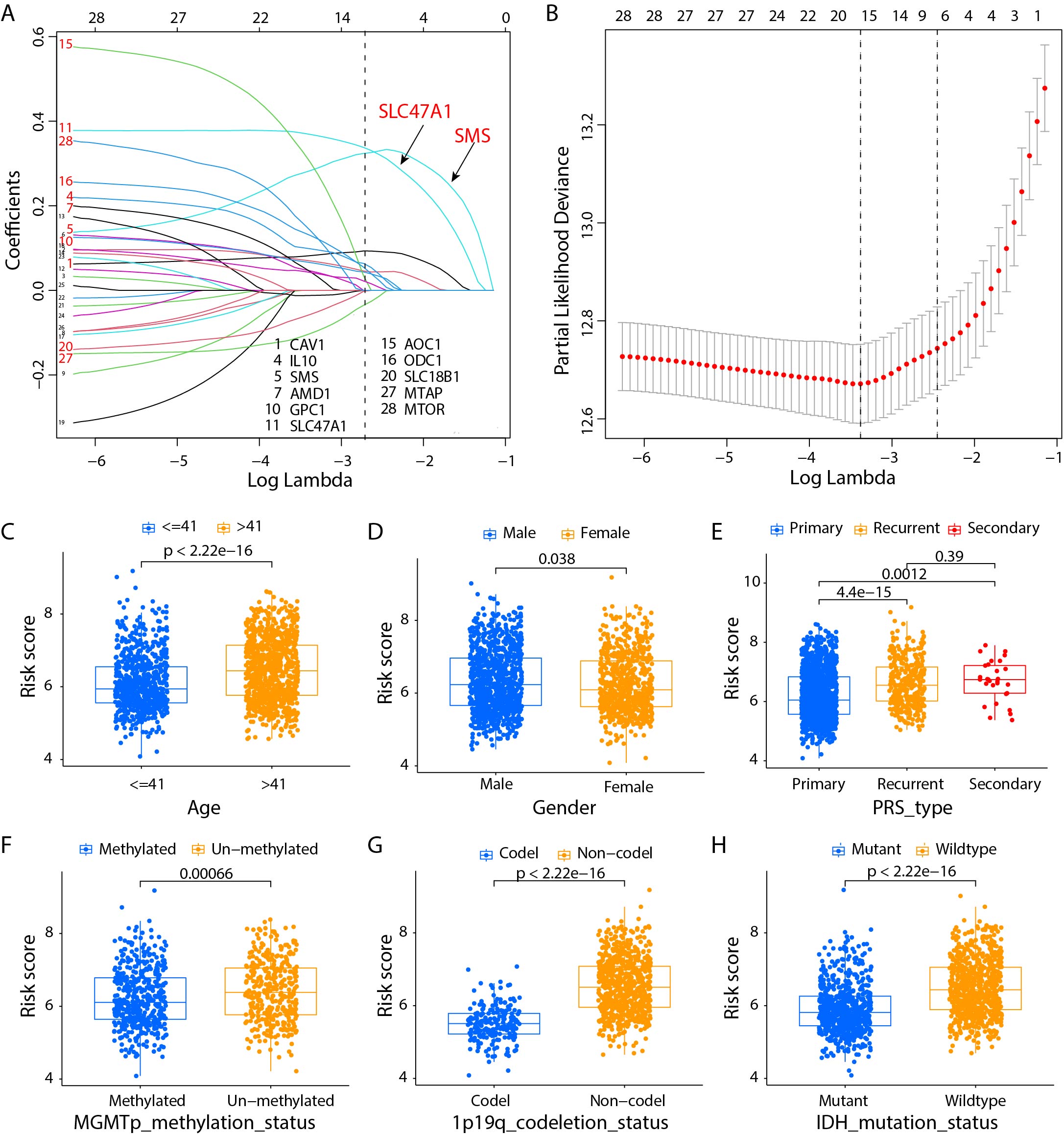

Supplement: Supplementary Figure 3 — (A, B) LASSO coefficient profiles of the 37 PMRGs. (C–H) The risk score for glioma patients based on their ages (C), gender (D), PRS types (E), MGMT methylation status (F), 1p19q codeletion status (G) and IDH mutation status (H). [file Image3.jpeg]

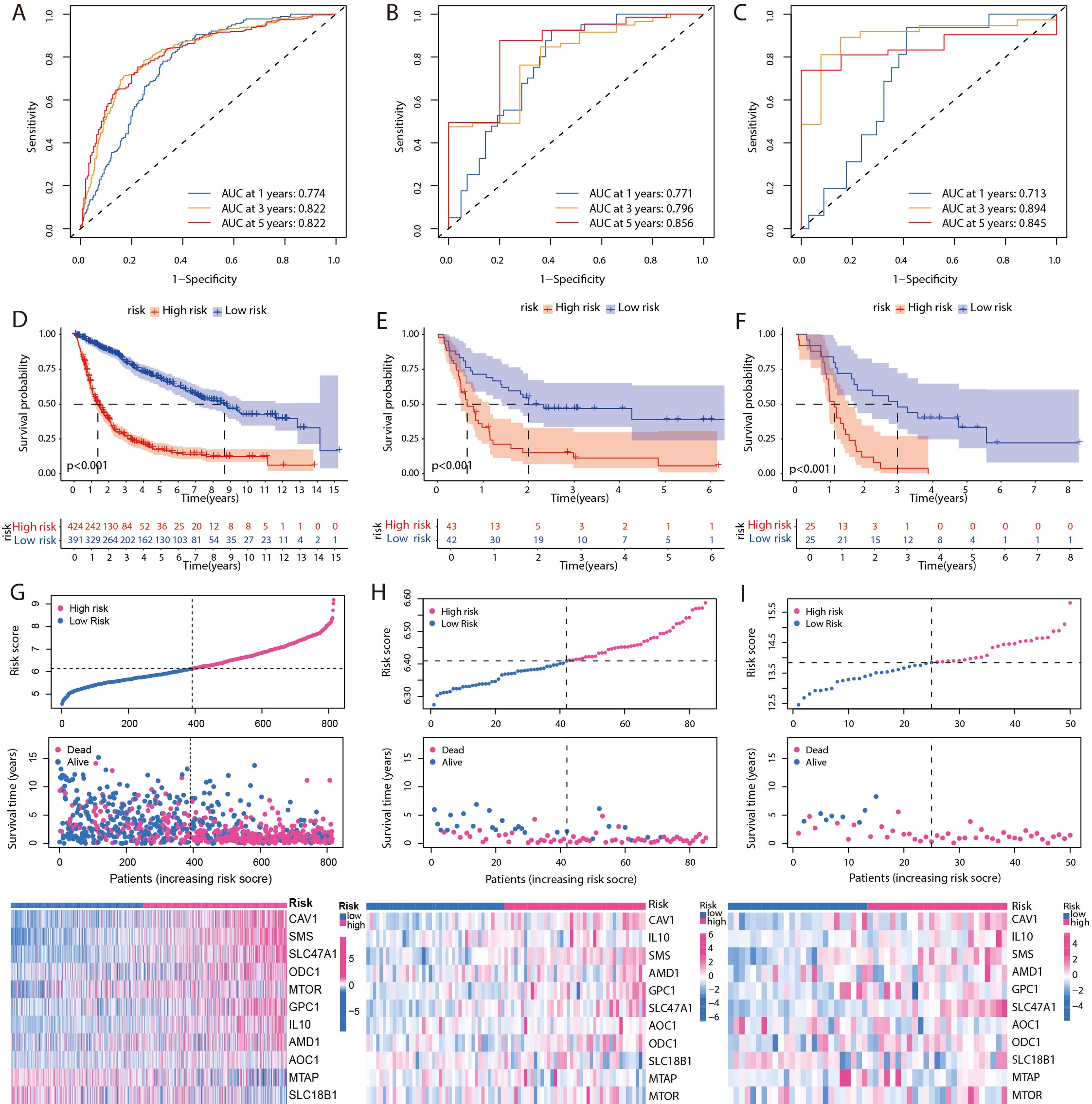

Supplement: Supplementary Figure 4 — Validation of the PMRG-related risk score in different cohorts. (A–C) ROC curves of the risk scores in the TCGA validation set, GSE4412, and GSE43378; (D–F) Kaplan–Meier curves of PMRG-related risk groups in the TCGA validation set, GSE4412, and GSE43378; (G–I) Risk score scatter plots of the TCGA validation set, GSE4412, and GSE43378. [file Image4.jpeg]

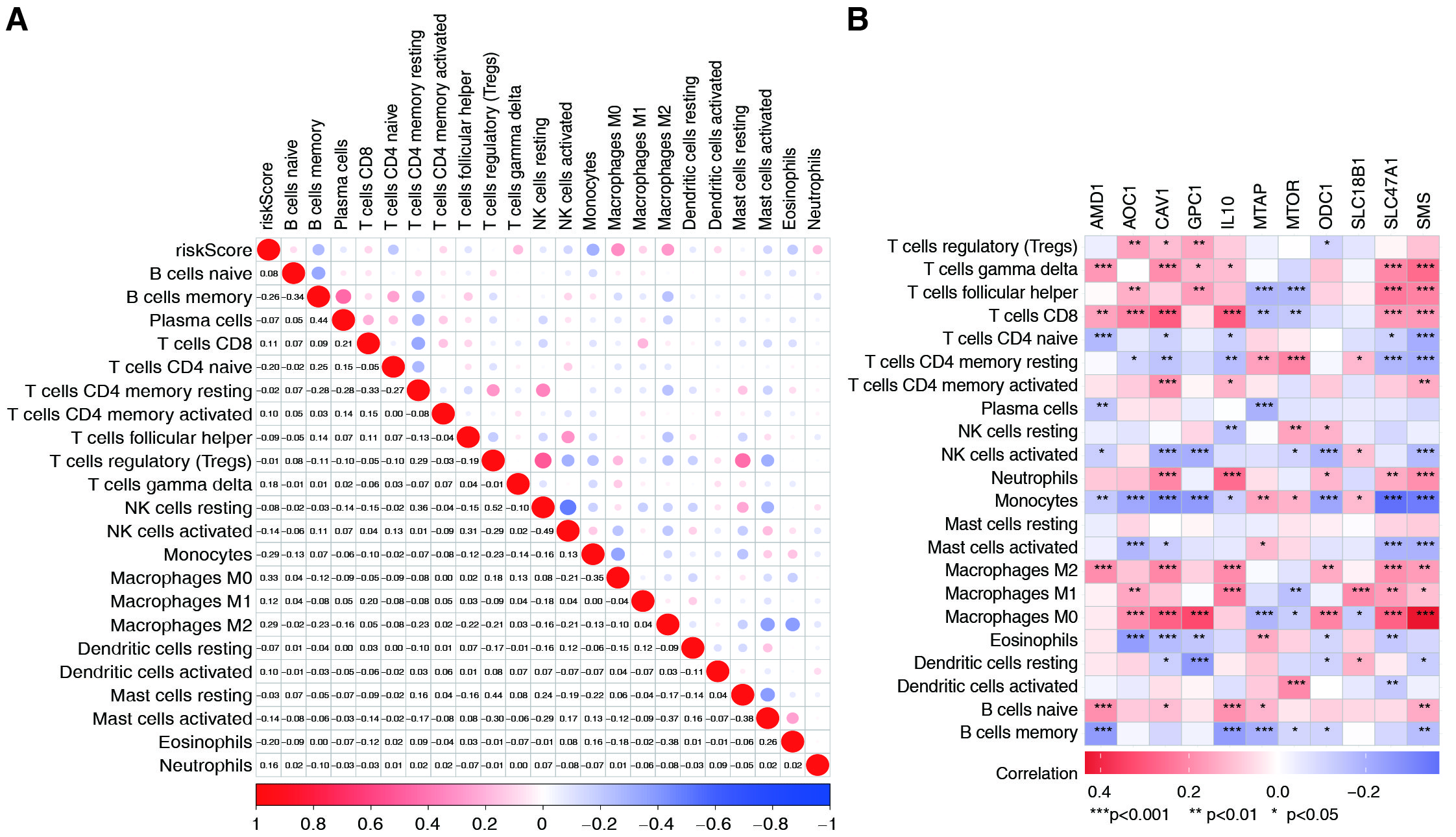

Supplement: Supplementary Figure 5 — Evaluation of immune cell infiltration based on PMRG-related risk subtypes. (A) Correlation matrix of 22 immune cell infiltrates with risk scores. (B) Association of five key PMRGs with immune cell infiltration. [file Image5.jpeg]
