# Supplementary material for: Integrative analysis of polyamine metabolism-related genes in gliomas: implications for prognosis and therapy
Source: Front Oncol. 2025 Jul 21;15:1517557. doi: 10.3389/fonc.2025.1517557 (PMC12319057; doi:10.3389/fonc.2025.1517557)
Supplement: Supplementary file 6 [file Table1.docx]

**Table S1. Clinical and molecular characteristics of patients included in TCGA and CGGA dataset.**

| **Cohort** | **CGGA-RNAseq**  **(n = 1038)** | **TCGA-RNAseq**  **(n = 672)** |
| --- | --- | --- |
| **Age, years** |  |  |
| Mean (range) | 43 (8 – 79) | 47 (14 – 89) |
| **Gender** |  |  |
| Female | 417 | 283 |
| Male | 601 | 384 |
| Unavailable | 0 | 5 |
| **Primary or recurrent** |  |  |
| Normal | 20 | 5 |
| Primary glioma | 651 | 641 |
| Recurrent glioma | 333 | 26 |
| Secondary GBM | 30 | 0 |
| Unavailable | 4 | 5 |
| **WHO Grade** |  |  |
| Normal | 20 | 5 |
| Grade II | 291 | 250 |
| Grade III | 334 | 262 |
| Grade IV | 388 | 155 |
| Unavailable | 5 | 0 |
| **Histology** |  |  |
| Normal | 20 | 5 |
| Oligodendroglioma | 112 | 114 |
| Oligodendroglioma, anaplastic | 94 | 79 |
| Astrocytoma | 175 | 63 |
| Astrocytoma, anaplastic | 214 | 128 |
| Oligoastrocytoma | 9 | 128 |
| Oligoastrocytoma, anaplastic | 21 | 0 |
| Glioblastoma | 388 | 155 |
| Unavailable | 5 | 0 |
| **IDH status** |  |  |
| Mutant | 531 | 126 |
| Wild-type | 435 | 296 |
| Unavailable | 72 | 250 |
| **1p/19q status** |  |  |
| Codel | 212 | 0 |
| Non-codel | 728 | 0 |
| Unavailable | 98 | 672 |
| **Overall survival, days** |  |  |
| Median | 1280 | 855 |
| **Survival status** |  |  |
| Alive | 362 | 408 |
| Dead | 617 | 256 |
| Unavailable | 59 | 8 |
