# Supplementary material for: Integrative analysis of polyamine metabolism-related genes in gliomas: implications for prognosis and therapy
Source: Front Oncol. 2025 Jul 21;15:1517557. doi: 10.3389/fonc.2025.1517557 (PMC12319057; doi:10.3389/fonc.2025.1517557)
Supplement: Supplementary file 7 [file Table2.docx]

**Supplementary Table 2**

**Table S2. Clinical characteristics of patients included in Glioma tissue microarray.**

| **Cat No.** | | ZL-BraG180sur01 | | | | |
| --- | --- | --- | --- | --- | --- | --- |
| **Species** | | ■human □rat □mouse □rabbit | | | | |
| **Thickness** | | □0.6mm □1.0mm ■1.5mm □2.0mm | | | | |
| **Description** | | 180 tumor specimens and 4 normal tissue | | | | |
| 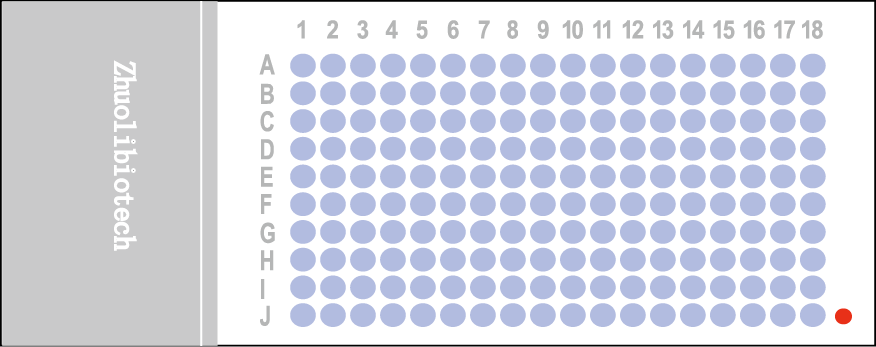 | | | | | | |
| **Position** | **Futime (month)** | **Vital status** | **Gender** | **Age** | **Grade** | **H-score(SMS)** |
|  |  |  |  |  | Normal | 79.1424 |
|  |  |  |  |  | Normal | 72.1234 |
|  |  |  |  |  | Normal | 73.7358 |
|  |  |  |  |  | Normal | 82.0514 |
| A01 | 30 | Alive | Male | 11 | G1 | 140.616 |
| A02 | 2 | Dead | Male | 17 | G1 | 171.629 |
| A03 | 13 | Dead | Male | 15 | G1 | 153.027 |
| A04 | 9 | Dead | Female | 8 | G2 | 144.717 |
| A05 | 4 | Dead | Male | 50 | G2 | 172.334 |
| A06 | 20 | Dead | Male | 43 | G2 | 112.036 |
| A07 | 19 | Dead | Male | 30 | G2 | 155.042 |
| A08 | 15 | Dead | Female | 49 | G2 | 158.359 |
| A09 | 22 | Dead | Male | 44 | G2 | 130.585 |
| A10 | 29 | Alive | Female | 48 | G2 | 157.192 |
| A11 | 19 | Dead | Female | 30 | G2 | 78.7774 |
| A12 | 14 | Dead | Female | 49 | G2 | 154.909 |
| A13 | 6 | Dead | Female | 36 | G2 | 145.349 |
| A14 | 12 | Dead | Male | 58 | G2 | 145.601 |
| A15 | 13 | Alive | Male | 27 | G2 | 165.651 |
| A16 | 21 | Dead | Female | 58 | G2 | 172.468 |
| A17 | 16 | Dead | Female | 52 | G2 | 135.234 |
| A18 | 18 | Dead | Male | 50 | G2 | 136.124 |
| B01 | 22 | Dead | Female | 45 | G2 | 172.507 |
| B02 | 38 | Dead | Female | 45 | G2 | 62.5289 |
| B03 | 29 | Dead | Male | 73 | G2 | 152.828 |
| B04 | 23 | Dead | Male | 36 | G2 | 186.424 |
| B05 | 14 | Dead | Male | 42 | G2 | 99.7251 |
| B06 | 22 | Dead | Male | 48 | G2 | 160.632 |
| B07 | 10 | Dead | Female | 52 | G2 | 145.099 |
| B08 | 11 | Dead | Female | 57 | G2 | 171.904 |
| B09 | 15 | Dead | Female | 41 | G2 | 147.785 |
| B10 | 4 | Dead | Female | 44 | G2 | 181.823 |
| B11 | 22 | Dead | Male | 29 | G2 | 154.486 |
| B12 | 2 | Dead | Male | 46 | G2 | 158.987 |
| B13 | 7 | Dead | Male | 43 | G2 | 87.5492 |
| B14 | 15 | Dead | Male | 30 | G2 | 187.922 |
| B15 | 12 | Dead | Male | 60 | G2 | 158.402 |
| B16 | 11 | Dead | Male | 64 | G2 | 176.971 |
| B17 | 8 | Dead | Male | 28 | G2 | 170.447 |
| B18 | 5 | Dead | Female | 50 | G2 | 129.484 |
| C01 | 1 | Dead | Male | 22 | G2 | 158.57 |
| C02 | 3 | Dead | Female | 34 | G2 | 154.343 |
| C03 | 7 | Dead | Female | 20 | G2 | 169.447 |
| C04 | 3 | Dead | Male | 26 | G2 | 143.831 |
| C05 | 7 | Dead | Female | 29 | G2 | 140.73 |
| C06 | 8 | Dead | Male | 61 | G2 | 163.261 |
| C07 | 24 | Dead | Female | 29 | G2 | 157.328 |
| C08 | 20 | Dead | Male | 46 | G2 | 159.891 |
| C09 | 14 | Dead | Male | 32 | G2 | 168.626 |
| C10 | 0 | Dead | Male | 39 | G2 | 142.461 |
| C11 | 8 | Dead | Male | 21 | G2 | 172.046 |
| C12 | 2 | Dead | Male | 72 | G2 | 160.756 |
| C13 | 4 | Dead | Male | 42 | G2 | 153.001 |
| C14 | 5 | Dead | Male | 68 | G2 | 121.699 |
| C15 | 13 | Dead | Female | 55 | G2 | 167.887 |
| C16 | 21 | Dead | Male | 43 | G2 | 164.389 |
| C17 | 14 | Dead | Male | 37 | G2 | 130.339 |
| C18 | 9 | Dead | Female | 45 | G2 | 145.849 |
| D01 | 8 | Dead | Female | 47 | G2 | 154.612 |
| D02 | 3 | Dead | Male | 35 | G3 | 171.762 |
| D03 | 7 | Dead | Female | 59 | G3 | 179.149 |
| D04 | 25 | Dead | Male | 41 | G3 | 172.436 |
| D05 | 29 | Dead | Male | 57 | G4 | 151.911 |
| D06 | 8 | Dead | Female | 64 | G3 | 123.404 |
| D07 | 14 | Dead | Female | 53 | G3 | 158.201 |
| D08 | 8 | Alive | Female | 40 | G3 | 170.659 |
| D09 | 8 | Dead | Male | 57 | G3 | 171.019 |
| D10 | 5 | Alive | Male | 54 | G3 | 154.77 |
| D11 | 1 | Alive | Male | 51 | G3 | 177.16 |
| D12 | 17 | Dead | Male | 22 | G3 | 156.94 |
| D13 | 7 | Dead | Female | 20 | G3 | 158.071 |
| D14 | 0 | Dead | Female | 41 | G3 | 139.153 |
| D15 | 9 | Dead | Male | 16 | G3 | 155.023 |
| D16 | 5 | Dead | Female | 40 | G3 | 154.939 |
| D17 | 16 | Dead | Female | 62 | G3 | 145.258 |
| D18 | 13 | Dead | Female | 41 | G3 | 166.007 |
| E01 | 26 | Dead | Female | 70 | G3 | 163.652 |
| E02 | 17 | Dead | Male | 50 | G3 | 172.815 |
| E03 | 16 | Dead | Female | 53 | G3 | 171.709 |
| E04 | 39 | Dead | Male | 54 | G3 | 167.825 |
| E05 | 7 | Dead | Male | 58 | G3 | 158.172 |
| E06 | 13 | Dead | Male | 39 | G3 | 179.043 |
| E07 | 13 | Dead | Male | 45 | G3 | 171.513 |
| E08 | 58 | Dead | Female | 女 | G3 | 113.157 |
| E09 | 16 | Dead | Female | 68 | G3 | 111.286 |
| E10 | 8 | Dead | Male | 27 | G3 | 132.017 |
| E11 | 27 | Dead | Female | 45 | G3 | 173.51 |
| E12 | 30 | Dead | Male | 27 | G4 | 195.795 |
| E13 | 13 | Dead | Male | 60 | G4 | 159.852 |
| E14 | 18 | Dead | Male | 67 | G4 | 158.341 |
| E15 | 10 | Dead | Female | 22 | G4 | 158.54 |
| E16 | 12 | Dead | Male | 57 | G4 | 160.35 |
| E17 | 23 | Dead | Male | 59 | G4 | 140.241 |
| E18 | 32 | Dead | Female | 73 | G4 | 175.303 |
| F01 | 6 | Dead | Female | 16 | G4 | 145.968 |
| F02 | 10 | Dead | Male | 64 | G4 | 177.099 |
| F03 | 8 | Dead | Female | 38 | G4 | 128.324 |
| F04 | 10 | Dead | Male | 56 | G4 | 185.346 |
| F05 | 14 | Dead | Female | 38 | G4 | 170.02 |
| F06 | 28 | Alive | Male | 57 | G4 | 163.004 |
| F07 | 21 | Dead | Male | 82 | G4 | 177.777 |
| F08 | 10 | Dead | Male | 31 | G4 | 167.695 |
| F09 | 18 | Dead | Female | 42 | G4 | 164.991 |
| F10 | 14 | Dead | Male | 54 | G4 | 158.612 |
| F11 | 16 | Dead | Male | 54 | G4 | 178.24 |
| F12 | 6 | Dead | Male | 57 | G4 | 158.085 |
| F13 | 16 | Alive | Male | 72 | G4 | 195.548 |
| F14 | 9 | Dead | Female | 41 | G4 | 162.888 |
| F15 | 13 | Dead | Female | 34 | G4 | 177.616 |
| F16 | 3 | Dead | Male | 74 | G4 | 160.433 |
| F17 | 2 | Dead | Male | 66 | G4 | 148.557 |
| F18 | 9 | Dead | Male | 72 | G4 | 174.545 |
| G01 | 9 | Dead | Male | 54 | G4 | 181.129 |
| G02 | 16 | Alive | Female | 68 | G4 | 150.668 |
| G03 | 13 | Dead | Male | 35 | G4 | 157.871 |
| G04 | 17 | Dead | Male | 66 | G4 | 165.624 |
| G05 | 5 | Dead | Female | 65 | G4 | 168.703 |
| G06 | 8 | Dead | Male | 54 | G4 | 175.914 |
| G07 | 14 | Dead | Male | 45 | G4 | 189.898 |
| G08 | 0 | Dead | Male | 74 | G4 | 168.754 |
| G09 | 3 | Alive | Female | 64 | G4 | 162.244 |
| G10 | 3 | Dead | Male | 76 | G4 | 166.999 |
| G11 | 1 | Alive | Female | 63 | G4 | 164.525 |
| G12 | 1 | Alive | Male | 56 | G4 | 161.817 |
| G13 | 20 | Dead | Female | 53 | G4 | 165.836 |
| G14 | 7 | Dead | Female | 64 | G4 | 175.058 |
| G15 | 17 | Dead | Female | 44 | G4 | 160.374 |
| G16 | 4 | Dead | Male |  | G4 | 164.258 |
| G17 | 11 | Dead | Male | 56 | G4 | 164.742 |
| G18 | 1 | Dead | Male | 52 | G4 | 149.189 |
| H01 | 5 | Dead | Male | 51 | G4 | 172.534 |
| H02 | 9 | Dead | Male | 70 | G4 | 171.225 |
| H03 | 4 | Dead | Female | 46 | G4 | 204.557 |
| H04 | 3 | Dead | Female | 78 | G4 | 166.015 |
| H05 | 9 | Dead | Male | 64 | G4 | 171.45 |
| H06 | 2 | Dead | Male | 50 | G4 | 171.828 |
| H07 | 2 | Dead | Male | 46 | G4 | 182.923 |
| H08 | 17 | Dead | Male | 55 | G4 | 168.682 |
| H09 | 6 | Dead | Male | 68 | G4 | 166.209 |
| H10 | 15 | Dead | Male | 77 | G4 | 185.367 |
| H11 | 6 | Dead | Female | 50 | G4 | 176.751 |
| H12 | 11 | Dead | Male | 67 | G4 | 164.502 |
| H13 | 4 | Dead | Male | 61 | G4 | 162.083 |
| H14 | 10 | Dead | Male | 58 | G4 | 127.421 |
| H15 | 1 | Dead | Male | 67 | G4 | 170.842 |
| H16 | 6 | Dead | Male | 56 | G4 | 148.202 |
| H17 | 6 | Dead | Female | 75 | G4 | 159.58 |
| H18 | 22 | Dead | Female | 43 | G4 | 170.758 |
| I01 | 10 | Dead | Male | 36 | G4 | 175.098 |
| I02 | 0 | Dead | Male | 78 | G4 | 172.974 |
| I03 | 16 | Dead | Male | 77 | G4 | 167.859 |
| I04 | 0 | Dead | Male | 73 | G4 | 186.416 |
| I05 | 7 | Dead | Female | 59 | G4 | 135.728 |
| I06 | 4 | Dead | Male | 58 | G4 | 171.147 |
| I07 | 5 | Dead | Female | 63 | G4 | 162.181 |
| I08 | 3 | Dead | Male | 67 | G4 | 171 |
| I09 | 0 | Dead | Female | 33 | G4 | 174.131 |
| I10 | 16 | Dead | Male | 43 | G4 | 174.986 |
| I11 | 8 | Dead | Female | 34 | G4 | 190.299 |
| I12 | 2 | Dead | Male | 49 | G4 | 163.747 |
| I13 | 3 | Dead | Male | 55 | G4 | 190.627 |
| I14 | 12 | Dead | Female | 74 | G4 | 169.334 |
| I15 | 16 | Dead | Male | 61 | G4 | 195.512 |
| I16 | 9 | Dead | Male | 48 | G4 | 177.131 |
| I17 | 13 | Dead | Male | 67 | G4 | 169.24 |
| I18 | 15 | Dead | Male | 53 | G4 | 168.81 |
| J01 | 30 | Dead | Male | 50 | G4 | 166.026 |
| J02 | 1 | Dead | Male | 65 | G4 | 170.448 |
| J03 | 12 | Dead | Female | 66 | G4 | 197.301 |
| J04 | 4 | Dead | Male | 68 | G4 | 182.92 |
| J05 | 2 | Dead | Male | 58 | G4 | 184.956 |
| J06 | 12 | Dead | Female | 68 | G4 | 180.202 |
| J07 | 28 | Dead | Male | 71 | G4 | 187.61 |
| J08 | 21 | Dead | Male | 62 | G4 | 146.794 |
| J09 | 2 | Dead | Female | 55 | G4 | 179.359 |
| J10 | 26 | Dead | Male | 59 | G4 | 161.484 |
| J11 | 19 | Dead | Female | 45 | G4 | 192.528 |
| J12 | 41 | Dead | Male | 55 | G4 | 167.096 |
| J13 | 21 | Dead | Male | 35 | G4 | 197.949 |
| J14 | 17 | Dead | Male | 51 | G4 | 173.917 |
| J15 | 27 | Dead | Male | 52 | G4 | 174.507 |
| J16 | 12 | Dead | Male | 64 | G4 | 200.226 |
| J17 | 0 | Dead | Male | 69 | G4 | 191.501 |
| J18 | 17 | Dead | Male | 50 | G4 | 183.303 |
| J19 | Positioning point | | | | | 210.992 |
