# Supplementary material for: Integrative analysis of polyamine metabolism-related genes in gliomas: implications for prognosis and therapy
Source: Front Oncol. 2025 Jul 21;15:1517557. doi: 10.3389/fonc.2025.1517557 (PMC12319057; doi:10.3389/fonc.2025.1517557)
Supplement: Supplementary file 8 [file Table3.docx]

**Supplementary Table 3**

**Table S3. Clinical characteristics of patients for validation of *SMS* mRNA.**

| **Type** | **Gender** | **Disease** |
| --- | --- | --- |
| Normal | Female | Traumatic brain injury |
| Normal | Male | Traumatic brain injury |
| Normal | Male | Traumatic brain injury |
| Tumor | Male | Glioma |
| Tumor | Male | Glioma |
| Tumor | Female | Glioma |
| Tumor | Male | Glioma |
| Tumor | Female | Glioma |
| Tumor | Female | Glioma |
