# Supplementary material for: Integrative analysis of polyamine metabolism-related genes in gliomas: implications for prognosis and therapy
Source: Front Oncol. 2025 Jul 21;15:1517557. doi: 10.3389/fonc.2025.1517557 (PMC12319057; doi:10.3389/fonc.2025.1517557)
Supplement: Supplementary file 9 [file Table4.docx]

**Table S4. List of 37 Polyamine-related genes.**

| **Gene** | **Protein names** | **Type** |
| --- | --- | --- |
| AGMAT | Agmatinase | Biosynthesis |
| AMD1 | S-adenosylmethionine decarboxylase proenzyme | Biosynthesis |
| ARG1 | Arginase-1 | Biosynthesis |
| ARNT | Aryl hydrocarbon receptor nuclear translocator | Biosynthesis |
| AZIN1 | Antizyme inhibitor 1 | Biosynthesis |
| AZIN2 | Antizyme inhibitor 2 | Biosynthesis |
| HIF1A | Hypoxia-inducible factor 1-alpha | Biosynthesis |
| IL10 | Interleukin-10 | Biosynthesis |
| IL1B | Interleukin-1 beta | Biosynthesis |
| IL4 | Interleukin-4 | Biosynthesis |
| MAT2B | Methionine adenosyltransferase 2 subunit beta | Biosynthesis |
| MTAP | S-methyl-5'-thioadenosine phosphorylase | Biosynthesis |
| MTOR | Serine/threonine-protein kinase Mtor | Biosynthesis |
| MYC | Myc proto-oncogene protein | Biosynthesis |
| ODC1 | Ornithine decarboxylase | Biosynthesis |
| SMOX | Spermine oxidase | Biosynthesis |
| SMS | Spermine synthase | Biosynthesis |
| SRM | Spermidine synthase | Biosynthesis |
| TNF | Tumor necrosis factor | Biosynthesis |
| AOC1 | Amiloride-sensitive amine oxidase | Catabolism |
| PAOX | Peroxisomal N(1)-acetyl-spermine/spermidine oxidase | Catabolism |
| RACK1 | Receptor of activated protein C kinase 1 | Catabolism |
| SAT1 | Spermidine/spermine N(1)-acetyltransferase 1 | Catabolism |
| ATP13A2 | Polyamine-transporting ATPase 13A2 | Transport |
| ATP13A3 | Polyamine-transporting ATPase 13A3 | Transport |
| CAV1 | Caveolin-1 | Transport |
| GPC1 | Glypican-1 | Transport |
| SLC12A8 | Solute carrier family 12 member 8 | Transport |
| SLC15A1 | Solute carrier family 15 member 1 | Transport |
| SLC18B1 | MFS-type transporter SLC18B1 | Transport |
| SLC22A1 | Solute carrier family 22 member 1 | Transport |
| SLC22A16 | Solute carrier family 22 member 16 | Transport |
| SLC22A2 | Solute carrier family 22 member 2 | Transport |
| SLC22A3 | Solute carrier family 22 member 3 | Transport |
| SLC2A1 | Solute carrier family 2, facilitated glucose transporter member 1 | Transport |
| SLC3A2 | Solute carrier family 3 member 2 | Transport |
| SLC47A1 | Solute carrier family 47 member 1 | Transport |
